# Heart Failure and MEF2 Transcriptome Dynamics in Response to β-Blockers

**DOI:** 10.1038/s41598-017-04762-x

**Published:** 2017-06-30

**Authors:** S. W. Tobin, S. Hashemi, K. Dadson, S. Turdi, K. Ebrahimian, J. Zhao, G. Sweeney, J. Grigull, J. C. McDermott

**Affiliations:** 1 Department of Biology, York University, Toronto, ON M3J 1P3 Canada; 2 Muscle Health Research Centre (MHRC), York University, Toronto, ON M3J 1P3 Canada; 3 Centre for Research in Biomolecular Interactions (CRBI), York University, Toronto, ON M3J 1P3 Canada; 4 Department of Mathematics and Statistics, York University, Toronto, ON M3J 1P3 Canada; 5 Centre for Research in Mass Spectrometry (CRMS), York University, Toronto, ON M3J 1P3 Canada

## Abstract

Myocyte Enhancer Factor 2 (MEF2) mediates cardiac remodelling in heart failure (HF) and is also a target of β-adrenergic signalling, a front-line treatment for HF. We identified global gene transcription networks involved in HF with and without β-blocker treatment. Experimental HF by transverse aortic constriction (TAC) in a MEF2 “sensor” mouse model (6 weeks) was followed by four weeks of β-blockade with Atenolol (AT) or Solvent (Sol) treatment. Transcriptome analysis (RNA-seq) from left ventricular RNA samples and MEF2A depleted cardiomyocytes was performed. AT treatment resulted in an overall improvement in cardiac function of TAC mice and repression of MEF2 activity. RNA-seq identified 65 differentially expressed genes (DEGs) due to TAC treatment with enriched GO clusters including the inflammatory system, cell migration and apoptosis. These genes were mapped against DEGs in cardiomyocytes in which MEF2A expression was suppressed. Of the 65 TAC mediated DEGs, AT reversed the expression of 28 mRNAs. *Rarres2* was identified as a novel MEF2 target gene that is upregulated with TAC *in vivo* and isoproterenol treatment *in vitro* which may have implications in cardiomyocyte apoptosis and hypertrophy. These studies identify a cohort of genes with vast potential for disease diagnosis and therapeutic intervention in heart failure.

## Introduction

Morbidity and mortality associated with cardiovascular disease (CVD) is a predominant global health problem occupying a prevalent position as the leading cause of death worldwide. Due to its universality, the multi-faceted progression of heart disease is therefore one of profound clinical importance. Progressive heart failure (HF), as one aspect of CVD, has a staggering prevalence of approximately thirty eight million diagnosed patients globally, a number which is growing due to the ageing population and the pervasiveness of HF in that age cohort^1^. Moreover, a HF diagnosis in many cases is an ailment with a poorer prognosis than most cancers^1^. Understanding and averting the progression of HF is therefore fundamental in the battle against CVD.

A significant body of cellular and molecular research has elucidated the many structural and signaling changes associated with HF, while current advances in transcriptional analysis have begun to unravel the underlying dysregulation of the cardiac transcriptome in the pathogenesis of CVD and heart failure. The regulation of the transcriptome in the heart is a primary determinant of its gene expression signature, phenotype and function^2–6^. Extensive work concerning the control of cardiac specific gene expression^7–12^ and also loss of function analysis in gene targeted mice^13–15^ has positioned Myocyte Enhancer Factor 2 transcriptional regulatory proteins (MEF2) at a nexus of control for cardiac and skeletal muscle gene expression. The MEF2 family of transcription factors (encoded by four genes labeled as MEF2A to D) have proved crucial in regulating cardiac^16^, skeletal^7, 17^ and smooth muscle differentiation^18^, neuronal survival and plasticity^19, 20^ and T cell activation^21^. The requirement for MEF2 is evolutionarily conserved for cardiac and skeletal muscle development from flies to humans^7^. Interestingly, transgenic mice that express a constitutively active CaMKIV gene exhibit cardiac hypertrophy and, when cross-bred with a MEF2 “sensor” mouse model, show markedly enhanced MEF2 activity^22^. There is further molecular and correlative data that MEF2 activity is enhanced during cardiac hypertrophy, but to date how this modulates the progression of disease *in vivo* is unclear. MEF2 is highly responsive to several signal transduction cascades, and post-translational regulation by covalent modification by PKC^23^, p38 MAPK^23–25^, ERK5^26, 27^ and PKA have been documented by ourselves^28^ and others^29, 30^.

Clinically, β-adrenergic receptor (βAR) antagonists, or β-blockers, reduce heart rate (chronotropy) and the force (inotropy) of myocardial contraction. Congestive heart failure in humans has been associated with impaired cardiac function, structural alteration, neurohormonal activation and the use of β-blockers in humans with heart failure results in decreased mortality^31, 32^. Despite the well characterized effects of β**-**adrenergic blockers in modulating cardiac inotropic and chronotropic parameters and improving overall cardiac function, there is a surprising lack of information concerning how acute and, particularly, chronic β**-**blocker treatment affects cardiac gene expression. To date, the impact of pharmacologic treatment by β-blockers, a first line HF treatment, on global transcription changes during HF has not been reported.

In view of the central role of MEF2 in the control of cardiac transcription we view MEF2 activity as a barometer of extensive transcriptional changes in the heart. Moreover, because of the potent role of β-adrenergic signalling in heart disease and its associated control of cardiac MEF2 activity, we postulated that this may be of potential relevance in the pathology of the heart. Therefore, we have begun to assess the relationship between global gene transcription and heart failure, particularly in response to β1-adrenergic blockade. Here, we report dynamic changes in cardiac gene transcription and MEF2 activity in response to HF, and document a subset of these genes that respond to pharmacologic manipulation by β1-adrenergic blockade.

## Results

### β-adrenergic blockade attenuates the hypertrophic response to TAC, enhances cardiac function and reduces MEF2 activity

**Figure 1:**
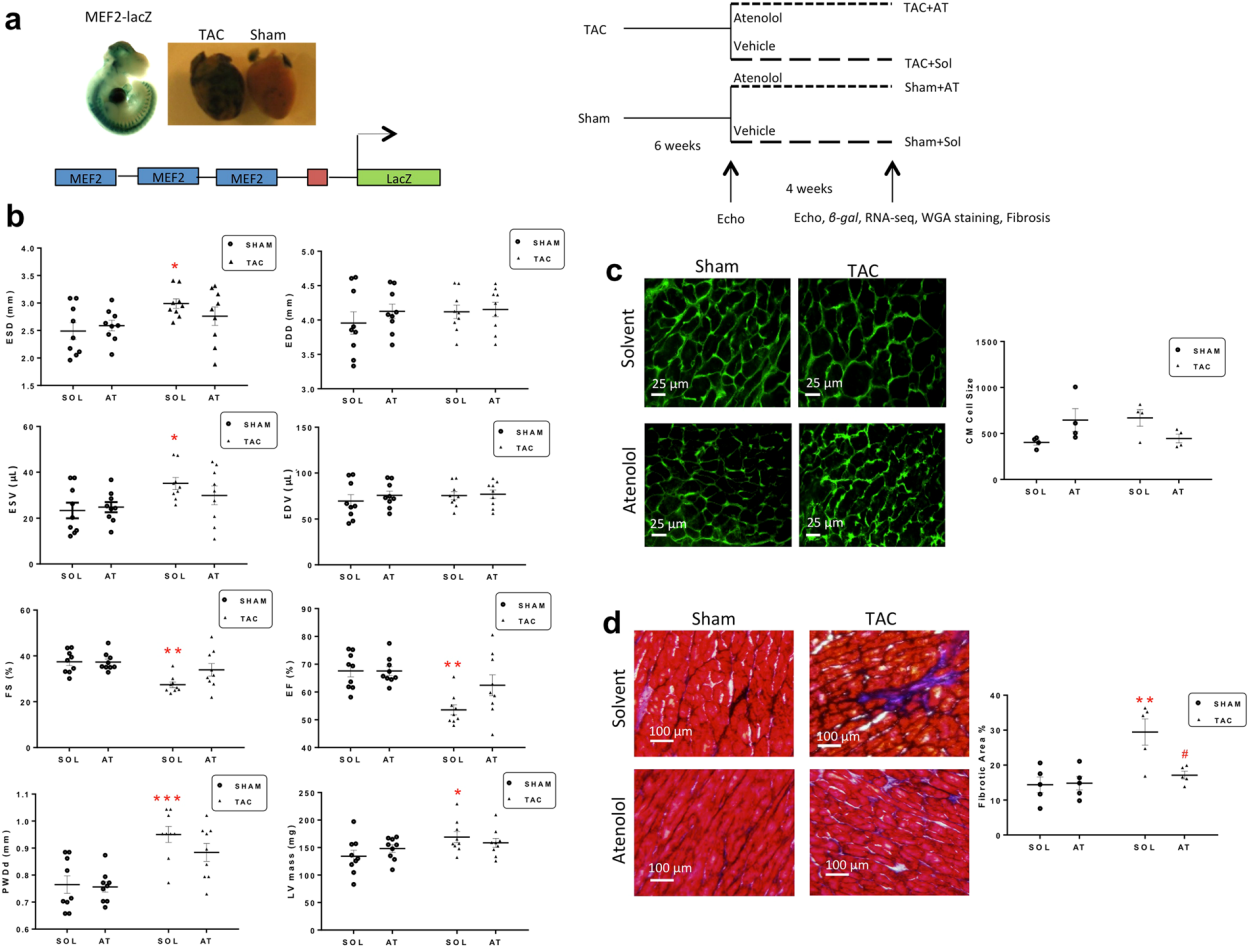
Atenolol reverses heart failure symptoms in TAC mice. (**a**) Experimental design overview. MEF2-LacZ mice (6–8 weeks old) underwent transverse aortic constriction (TAC) or Sham surgeries, followed by Solvent or Atenolol (50 mg/kg/day) treatment (n = 9). (**b**) Functional analysis of TAC or Sham mouse hearts after four weeks of Atenolol or Solvent treatment. End systolic diameter (ESD), end diastolic diameter (EDD), ejection fraction (EF), fractional shortening (FS), end systolic volume (ESV), end diastolic volume (EDV), left ventricular mass (LV), and left ventricular posterior wall depth (PWDd) were assessed by ultrasound echocardiography. (n = 9), Data are presented as mean ± SEM. *P < 0.05 **P < 0.01 ***P < 0.001 vs Sham + Sol. (**c**) WGA stain depicting cardiac hypertrophy. Images are representative of an average of n ≥ 10 images per heart, 4 animals per group. Scale bar: 25 µm. Graph below shows cardiomyocyte size (CM cell size), quantified based on an average of ≥10 cell measurements per mouse (n = 4). (**d**) Masson’s Trichrome stain depicting fibrosis. Images are representative of n = 10 images per heart, 5 animals per group. Cardiomyocytes appear red, nuclei appear black and collagen appears blue. Scale bar: 100 µm. Graph shows quantified cardiomyocyte fibrosis based on a three representative images per mouse (n = 5). Data are presented as mean ± SEM. **P < 0.01 vs Sham + Sol. ^#^P < 0.05 vs TAC + Sol.

Our initial purpose was to determine the effects of β-blockade on MEF2 activity and cardiac function. To do this we used Transverse Aortic Constriction (TAC) which is a widely used *in vivo* model that mimics disease associated with cardiac hypertrophy and heart failure^33^. The β1 selective β-adrenergic antagonist, Atenolol was used in these experiments. Since MEF2 regulates a large battery of cardiac genes we were intrigued to further pursue these observations since it may have important clinical relevance for understanding the effects of chronic β-blocker treatment. To study the effects of TAC and chronic treatment with Atenolol on MEF2 activity we utilized a MEF2-LacZ reporter transgenic mouse model. These mice harbour a synthetic transgene containing three MEF2 sites from the Desmin gene distal to a heterologous promoter and exhibit MEF2 activity during development in the heart and somites^34^. Similar to previous studies with CAMKII activation^22^, we observed that MEF2 activity is markedly enhanced by TAC during cardiac hypertrophy (Fig. 1a) as we reported previously^35^. In contrast, long term Atenolol (>10 weeks) treatment reduced MEF2-activity in non-treated mice (Supplementary Fig. S1). Mice at 6–8 weeks of age were randomized to receive either TAC surgery to induce left ventricular (LV) pressure overload, or a sham operation. Assessment of heart function by transthoracic echocardiography six weeks after surgery demonstrating that TAC mice had developed physiological readouts characteristic of cardiac hypertrophy and the onset of heart failure: increased LV posterior wall thickness (PWDd) and LV mass, and decreased ejection fraction (EF) and fractional shortening (FS) (Supplementary Fig. S2, p < 0.05). 6 weeks after TAC or sham operations, mice were then treated with Solvent (Sol) or the β-blocker Atenolol (AT; 50 mg/kg/day) for four additional weeks. After which time a series of comprehensive physiological, biochemical and genomic analyses were carried out. The four experimental conditions were therefore: Sham + Sol; Sham + AT; TAC + Sol; and TAC + AT (Fig. 1a). The mice in our study were not in end stage heart failure, this is likely because the TAC is minimally invasive and to some degree variable. The conditions used in our study were consistent with those reported by Faerber *et al*.^36^ in which 7 weeks after minimally invasive TAC approximately 60% of the mice displayed signs of heart failure such as pleural effusions, dyspnea and contractile dysfunction. We therefore contend, based on our physiologic data in comparison with those in the literature^36^ that the TAC treatment in our study is indicative of the progression to end stage heart failure and, as such, depicts moderate heart failure and not end stage failure with a dilated phenotype.

After 4 weeks of Sol treatment, TAC + Sol mice continued to exhibit signs of cardiac dysfunction and LV hypertrophy: reduced EF and FS, and increased PWDd, end systolic diameter (ESD), end systolic volume (ESV) and LV mass compared to Sham + Sol (Fig. 1b, p < 0.05). End diastolic diameter (EDD) and volume (EDV) were similar between the TAC + Sol and Sham + Sol groups. Atenolol treatment in the sham group (Sham + AT) induced no statistically significant change in heart function when compared to the control group (Sham + Sol). However, four weeks of Atenolol treatment in the TAC group (TAC + AT) resolved changes to LV geometry and reversed indices of LV hypertrophy induced by pressure overload as indicated by a reduction in ESD, PWDd and LV mass compared to TAC + Sol, and this was associated with an overall improvement in cardiac function, as indicated by an increase in FS and EF in TAC + AT compared to TAC + Sol (Fig. 1b).

To further document the changes occurring in TAC mice treated with Atenolol, tissue sections were stained for wheat germ agglutinin (WGA) to visualize and quantitate cellular hypertrophy (Fig. 1c). TAC + Sol had larger cardiomyocytes than control. Cardiomyocyte surface area in the TAC + AT group was smaller than in the TAC + Sol group although that change was not statistically significant, therefore indicating a trend towards a reversal of the cardiomyocyte hypertrophic phenotype. We also noted that AT + Sham treatment enhanced cardiomyocyte size which is a somewhat confounding result that is not explicable at this time. One might argue that treating healthy animals with high level β-adrenergic blockade may be abnormal since the heart is not stressed nor under pathological load. It is highly speculative but under such conditions this response might result from depression of normal cardiac function but this requires further characterization. It is clear that AT treatment alone caused substantial changes in the transcriptome.

TAC and LV pressure overload is also known to be associated with increased collagen deposition leading to fibrosis, resulting in increased wall stiffness and ultimately decreased cardiac function^35, 37^. Masson’s trichrome staining showed increased fibrosis in the TAC + Sol group (p < 0.01) and this was reduced in the TAC + AT group, as shown in representative images in Fig. 1d (p < 0.05). Together, these data demonstrate positive effects of chronic Atenolol treatment on physiological and structural parameters associated with cardiac hypertrophy and heart failure progression.

### Dynamic changes in gene expression provoked by TAC are reversed by β-adrenergic blockade

**Figure 2:**
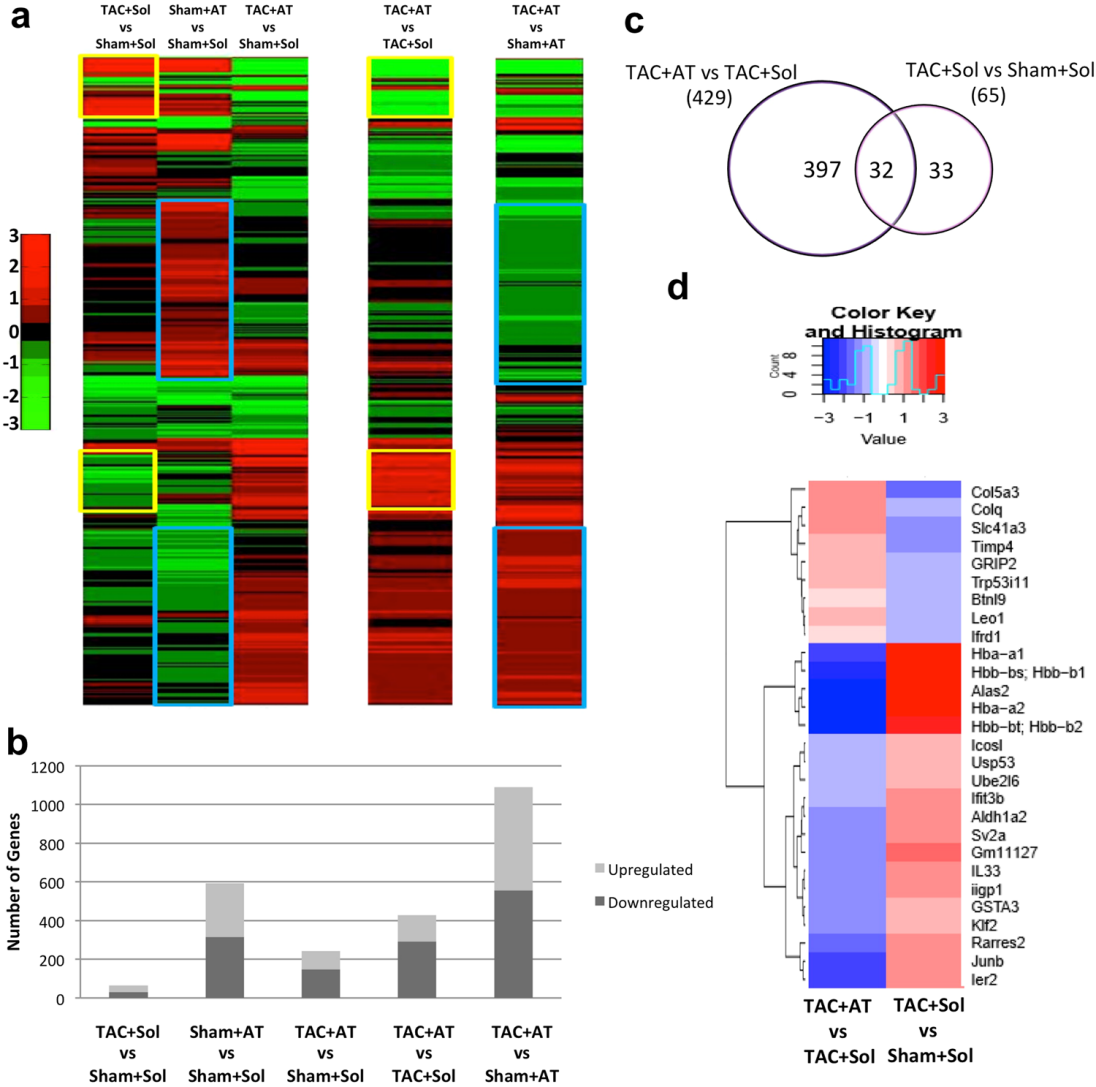
Changes in gene expression caused by aortic constriction is reversed by atenolol treatment. Four weeks after Atenolol treatment of TAC or Sham operated mice, RNA-seq analysis of whole tissue from the left ventricle was performed. Similar tissue was collected the Solvent control group (n = 3 per group). (**a**) A heatmap depicting changes in gene expression of 1571 protein coding genes. Genes are ordered based on hierarchical clustering. Genes up- or down-regulated by TAC alone, relative to control (leftmost column; yellow box). Effects of β-blocker (2^nd^ leftmost column; blue box). (**b**) The total number of up and downregulated genes in each condition. (**c**) A select group of genes (32) were differentially regulated in both the TAC + Sol vs Sham + Sol and TAC + AT vs TAC + Sol conditions. (**d**) A heatmap depicting the Fold Change values of TAC + AT vs TAC + Sham and TAC + Sol vs Sham + Sol.

To further understand the underlying changes in gene expression during these conditions, the left ventricle was isolated from each condition (n = 3 mice per condition) and RNA was prepared for subsequent RNA-Seq analysis to document transcriptome changes associated with the experimental treatments and potentially identify differentially expressed genes (DEGs). Across all conditions, 1571 DEGs were detected (Fig. 2a). In particular, we documented 65 DEGs in TAC + Sol mice compared to Sham + Sol (Fig. 2b). In parallel, some genes affected by Sham + Sol had a reversed pattern of expression in TAC + AT (Fig. 2a, blue boxes). A complete list of all DEGs is in Dataset S1. Most importantly, one predominant feature of the data is that the pattern of differentially expressed genes in TAC + Sol was in general reversed in TAC + AT (Fig. 2a, yellow boxes).

To identify the potentially most biologically relevant DEG’s we focused on genes that were differentially expressed in both TAC + Sol vs Sham + Sol and TAC + AT vs TAC + Sol comparisons (32 genes), which comprised of nearly half of all DEGs in the TAC + Sol condition (Fig. 2c). Figure 2d depicts the dynamic regulation of a sub-set of the 32 genes identified in Fig. 2c. In general, genes that were upregulated in TAC + Sol relative to Sham + Sol were downregulated in TAC + AT vs TAC + Sol, and vice versa for downregulated genes, demonstrating that Atenolol reverses the effect of TAC on gene expression in the heart. Among these genes were *Klf2*, *Junb*, *Ier2* (immediate early response 2), *A*l*as2* (delta-aminolevulinic acid synthase 2, two ubiquitin related proteins *Ube2l6* (E2 ubiquitin ligase) and *Usp53* that are upregulated in TAC, while Atenolol reduces their expression in response to TAC. The upregulation of α and β chains of hemoglobin seen in TAC + Sol were also reversed by Atenolol treatment, an important feature since elevated hemoglobin has been associated with cardiac death^38^. On the other hand *Ifrd1* (also known as PC4; Tis7) is a known co-factor of MEF2 in skeletal myoblasts that contributes to skeletal myogenesis^39^ is downregulated with TAC, but atenolol treatment induces expression relative to TAC + Sol. Down-regulation of tissue inhibitors of metalloproteinases (Timps) and upregulation of matrix metalloproteinases is also associated with extracellular matrix remodelling during heart failure^40, 41^. In our data, *Timp*-*4*, was downregulated with TAC and upregulated with Atenolol treatment. *Timp*-*4* downregulation has also been associated with left ventricular dilatation in heart failure in humans^42^. It is important to point out that our analysis isolated poly-A containing transcripts, so any lncRNAs without this modification would not be detected in our analysis. However, our analysis did indicate that there are 84 differentially expressed lncRNA across all conditions (Supplementary Fig. S3 and Dataset S2).

**Figure 3:**
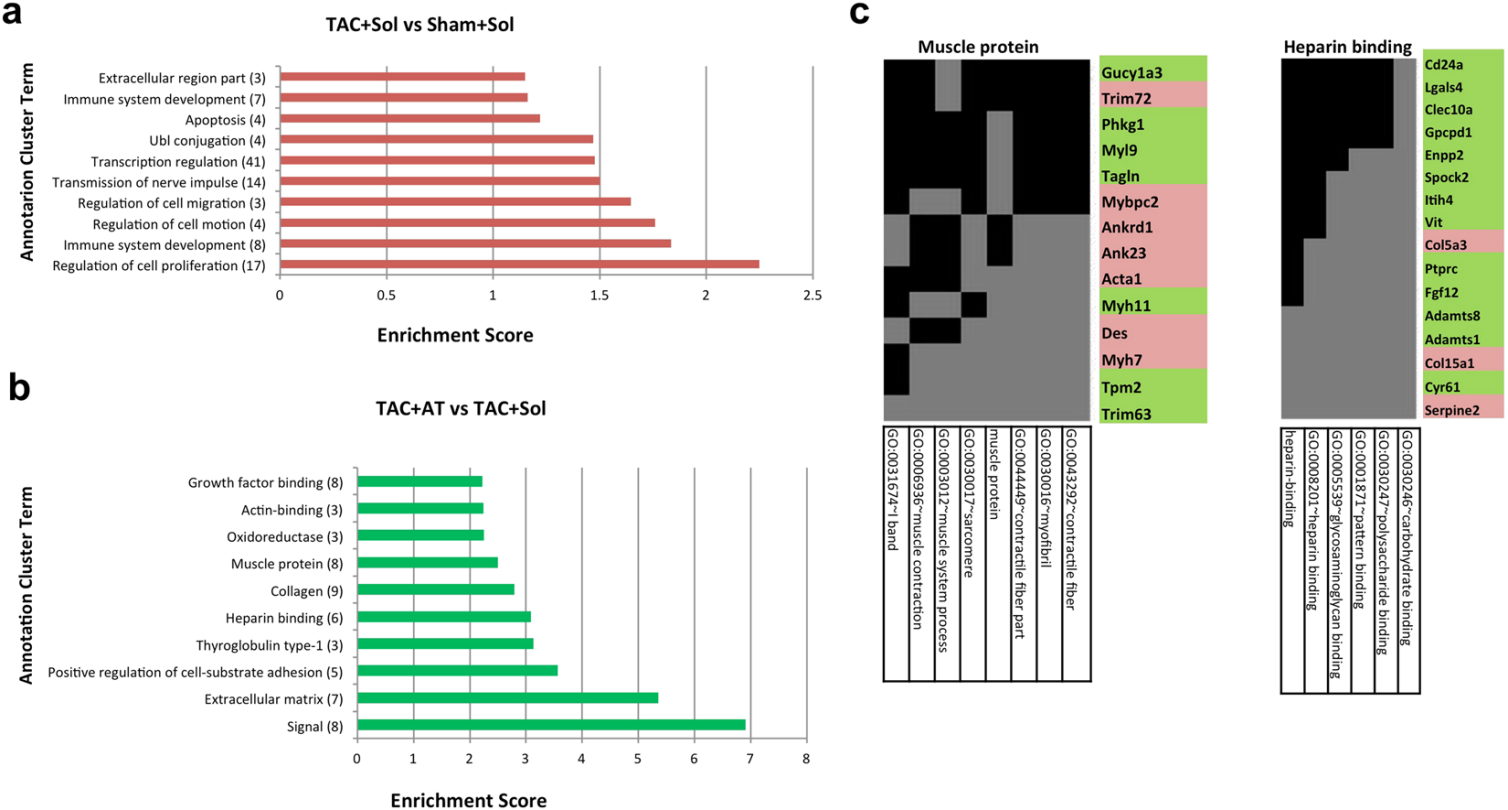
Biological implications based on functional annotation gene clustering. Function annotation clusters identified by DAVID of differentially expressed genes between (**a**) TAC + Sol and Sham + Sol or (**b**) TAC + AT vs TAC + Sol. Annotation clusters were identified using default settings with medium stringency. Beside each annotation is the number of representative terms associated within each cluster. (**c**) Two representative clusters from TAC + AT vs TAC + Sham. Representative terms within each cluster are on the X axis. Genes within each GO term are on the Y axis. Left: ‘Muscle protein’ (7 genes up, 7 down, 14 total). Right ‘Heparin binding’ (3 up genes, 13 down, 16 total). Grey indicates association of a gene with a representative term. Genes shaded red or green were up- or down-regulated, respectively.

We used a functional annotation clustering tool^43, 44^ to group terms with related biological meaning into annotation clusters. The enrichment score of each annotation cluster is the -log geometric mean of p-values of each annotation term within the cluster (eg. an enrichment score of >1.3 is equivalent to non-log scale p < 0.05). Therefore the higher the enrichment score, the more likely these annotations have a biologically relevant role. Annotation terms were manually selected from the list of representative terms generated within each cluster. The ten clusters with the highest enrichment score from each treatment are shown in Fig. 3. TAC + Sol (Fig. 3a) contained clusters enriched for cell proliferation, the immune system, cell migration and apoptosis. The most critical comparison was between TAC + Sol and TAC + AT (Fig. 3b). Annotation clusters were related to the extracellular matrix, collagen and heparin binding, and also muscle protein. To draw out a more gene specific comparison, two representative GO clusters from Fig. 3b are illustrated in Fig. 3c corresponding to the ‘muscle protein’ (left) and ‘heparin binding’ (right) identifiers. Terms within each annotation are on the X axis, while each gene associated with these terms is on the Y axis. Genes that were upregulated are red and genes that were downregulated are green. Fourteen genes were associated within the ‘muscle protein’ cluster. Some upregulated genes of note include *Mybpc2*, *Myh7*, *Acta1* and *Des*. Interestingly two of the affected genes were E3 ubiquitin ligases *Trim63* and *T*r*im72*. *Trim63*, (also called MuRF1) was downregulated and has been previously associated with muscle atrophy^45^. *Trim72* (or MG53) was upregulated and is associated with membrane stability has been reported to target the insulin receptor for degradation^46^. In general genes associated with the heparin binding cluster were downregulated in TAC + AT compared to TAC + Sol. Consistent with the high glycosaminoglycan binding properties of the protein products of these genes, several genes within this cluster are related to the extracellular space. Ccn1 (gene name in Fig. 3 is Cyr61) is a matricellular protein whose expression has been associated with heart failure^47^. Mice deficient for Col15a1 (type XV collagen) also have heart defects^48, 49^. Adamts1 and 8, two matrix metalloproteinases, are also known to be anti-angiogenic^50^. Precise expression of Adamts1 during embryogenesis is involved with trabeculation of the heart but its role in the adult heart is not yet clear^51^.

In general, these data demonstrate that during TAC + Sol treatment genes associated with apoptosis and remodeling of the extracellular matrix are upregulated and Atenolol treatment remarkably reverses many of these transcriptome changes. In addition, TAC + AT resulted in an upregulation of muscle protein encoding genes that are collectively associated with improved muscle function.

### The role of *Klf2*, *Junb*, *Rarres2* and *Alas2* in cardiomyocyte hypertrophy and apoptosis

**Figure 4:**
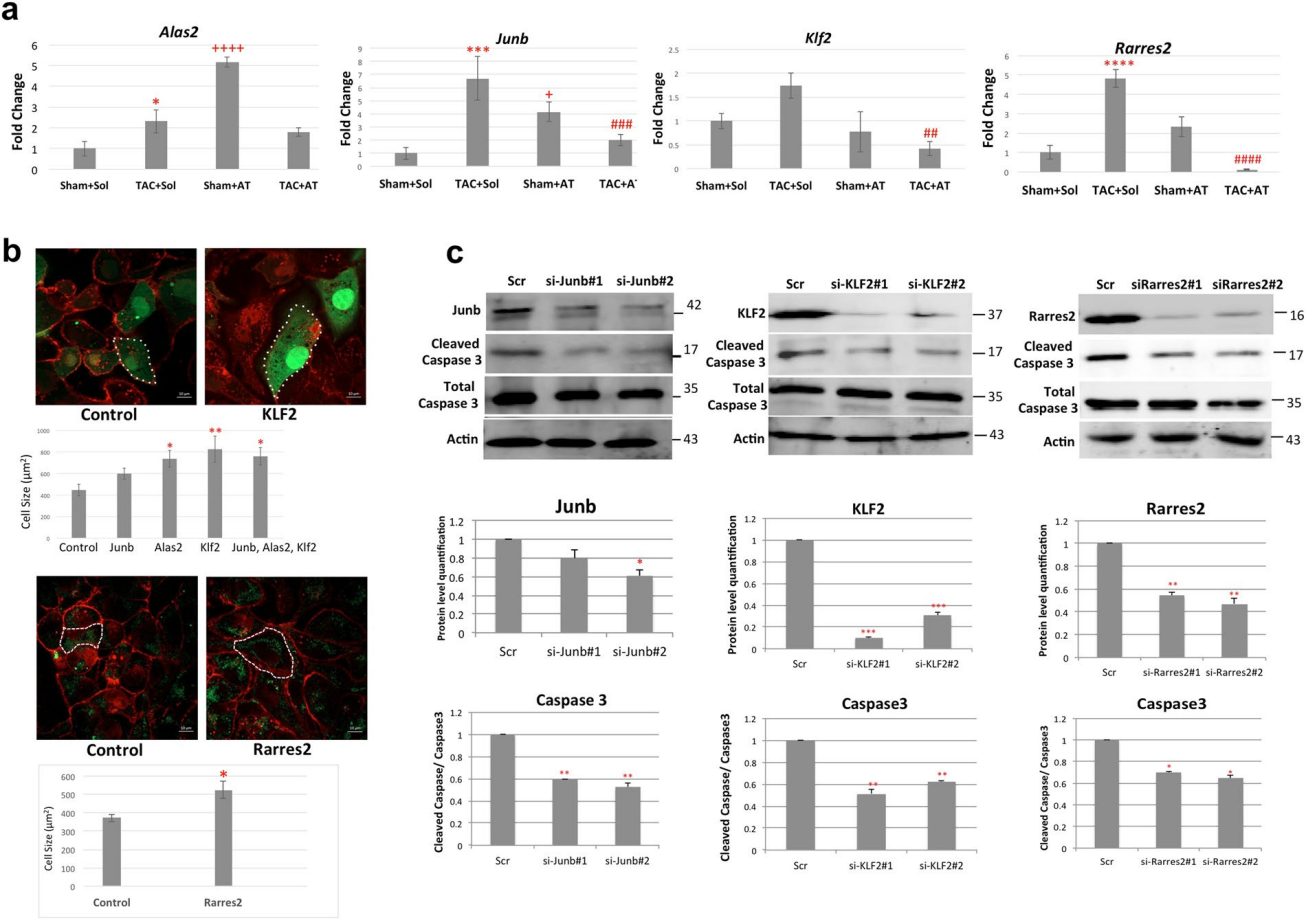
Role of Klf2, Junb, Rarres2 and Alas2 in cardiomyocyte hypertrophy and apoptosis. (**a**) Modulation of *Klf2*, *Junb*, *Rarres2* and *Alas2* expression in response to TAC and Atenolol as depicted in Fig. 2c, was confirmed using RT-qPCR. cDNA was analyzed via RT-qPCR using SYBR Green master mix. Data were normalized to Gapdh and are presented as fold change using the delta delta Ct method (n = 3, *P < 0.05 ***P < 0.001 ****P < 0.0001 Sham + Sol vs TAC + Sol, ^+^P < 0.05 ^++++^P < 0.0001 Sham + Sol vs Sham + AT, ^##^P < 0.01 ^###^P < 0.001 ^####^P < 0.0001 TAC + Sol vs TAC + AT). (**b**) WGA staining depicts cardiac hypertrophy. Cardiac HL-1 cells (upper panel) were transfected with GFP and Alas2, Junb, or Klf2 expression plasmids individually or in a mixture and stained with Wheat Germ Agglutinin (WGA) in red. The bar graph indicates that cell size of GFP positive HL-1 cells, quantified based on 5 cell measurements per image. Data are presented, as mean ± SEM. *P < 0.05 **P < 0.01 vs control. Scale bar is 10 μm. Cardiac HL1 cells (bottom panel) were transfected with GFP and Rarres2 expression plasmids and stained with WGA in Red. The bar graph indicates cell size of GFP positive HL-1 cells, quantified based on 10 cell measurements per image. Data are presented, as mean ± SEM. *P < 0.05 vs control. Scale bar is 10 μm. (**c**) Primary cardiomyocytes were transfected with two independent siRNAs targeting Junb, Rarres2 or Klf2 and then treated with isoproterenol (10 μM) for 48 hours. A scrambled siRNA was used as a control (Scr). The expression of cleaved Caspase 3 was assessed to determine changes in cardiomyocyte apoptosis using western blot analysis. A bar graph below each western blot represents the level of Cleaved Caspase 3 protein expression after normalized to total Caspase 3 (bottom panel). Data are presented as mean ± SEM. n = 3 *P < 0.05 **P < 0.01 ***P < 0.001 vs control. The western blots are cropped. An odyssey quantitative blotting system is used for the western blot analysis, therefore the quantitation is linear.

To verify the differential regulation of individual genes in response to TAC and Atenolol, as depicted in Fig. 2c and d in the transcriptomics data, we performed individual RT-qPCR on RNA samples from the same treatment conditions on 24 of the identified genes (Fig. 4a, Supplementary Fig. S4, S5). Next, we selected some genes that were upregulated in TAC + Sol relative to Sham + Sol and downregulated in TAC + AT vs TAC + Sham (*Klf2*, *Junb*, *Rarres2*, *Iigp1* and *Alas2*) for further analysis. The rationale for choosing these particular genes was that they were dysregulated under TAC conditions and, importantly, their expression pattern was substantially reversed with Atenolol treatment indicating that they may be involved in pathological changes that can be potentially altered by drug therapy. Since two prominent features of progressive heart failure are cardiomyocyte hypertrophy and apoptosis, we attempted to further test the capacity of some of the identified genes in *vitro* using gain and loss of function analysis. For the gain of function (GOF) analysis we chose to exogenously *Klf2*, *Junb*, *Alas2* alone and in combination to assess their effects on induction of hypertrophy in the cardiac HL-1 cell line. In a subsequent experiment we also assessed the role of *rarres 2 in this context*. In terms of apoptosis induction we reasoned that the genes identified might be pro-apoptotic since they are elevated with TAC and downregulated by AT treatment in the TAC model. Therefore, to assess this we tested loss of function (LOF) of *Klf2*, *Junb*, *Rarres2*, *Iigp1* and *Alas2* gene products using siRNA mediated depletion under conditions of apoptotic induction by high level Isoproterenol (Iso) treatment in primary cardiomyocytes. The readout used for apoptotic induction was the ratio of cleaved Caspase 3 to total Caspase 3. First, RT-qPCR analysis confirmed that *Klf2*, *Junb*, *Rarres2*, and *Alas2* are upregulated in response to TAC, and Atenolol treatment reverses this effect (Fig. 4a) similar to what was observed with TAC and Atenolol treatment *in vivo* in the transcriptome data (Fig. 2). Next, cardiac HL-1 cells were transfected with GFP and expression plasmids for a number of genes of interest individually or in combination and the cells were stained for Wheat Germ Agglutinin (WGA) to visualize and quantitate the cell surface area as an index of cellular hypertrophy (representative images are included in Fig. 4b). Only GFP+ cells were quantified for this analysis. The bar graph (upper panel) indicates the mean size of HL-1 cells transfected with the indicated expression plasmids individually or in combination compared to the vector alone control and the bar graph (bottom panel) shows the mean size of HL1 cells transfected with *Rarres2* expression plasmid compared to the corresponding control. These preliminary data indicate that, of the genes tested, *Alas2*, *Junb, Klf2* and *Rarres2* may have a role in promoting cardiomyocyte hypertrophy since their exogenous expression in cultured HL-1 cells led to a statistically significant enhancement in cell surface area with *Rarres2*, *Alas2*, or *Klf2* alone. A combination of *Alas 2, Jun B* and *Klf2* also resulted in enhanced cell size (Fig. 4, p < 0.05).

To assess the potential role of *Junb*, *Klf2*, *Rarres2*, *Alas2* and *Iigp1* genes on induction of cardiomyocyte apoptosis, we silenced their expression using siRNA technology and treated cardiomyocytes with isoproterenol (10 μM) for 48 hours to induce increased levels of cardiac apoptosis as we have previously published^52^. Of the genes tested Caspase 3 activation was reduced in siRNA-mediated depletion of *Junb*, *Rarres2* and *Klf2* treated with Iso compared to the control (Fig. 4c, p < 0.05) but depletion of *Alas2* or *Iigp1* had no effect (Supplementary Fig. S7). These data demonstrate a possible pro-apoptotic role of *Junb*, *Rarres2* and *Klf2* genes in primary cardiomyocytes.

In summary, these preliminary *in vitro* data concerning the possible function of the genes identified in the *in vivo* transcriptomics data reveal a putative involvement of *Junb*, *Rarres2* and *Klf2* in apoptotic induction and a potential hypertrophic influence of *Alas2*, *Junb*, *Klf2* and *Rarres2* in hypertrophy. Further *in vivo* studies of these genes are thus warranted to determine their role in cardiac pathology.

### Transcriptome profiling in MEF2A depleted cardiomyocytes and its relation to β-blockade associated transcriptome changes

**Figure 5:**
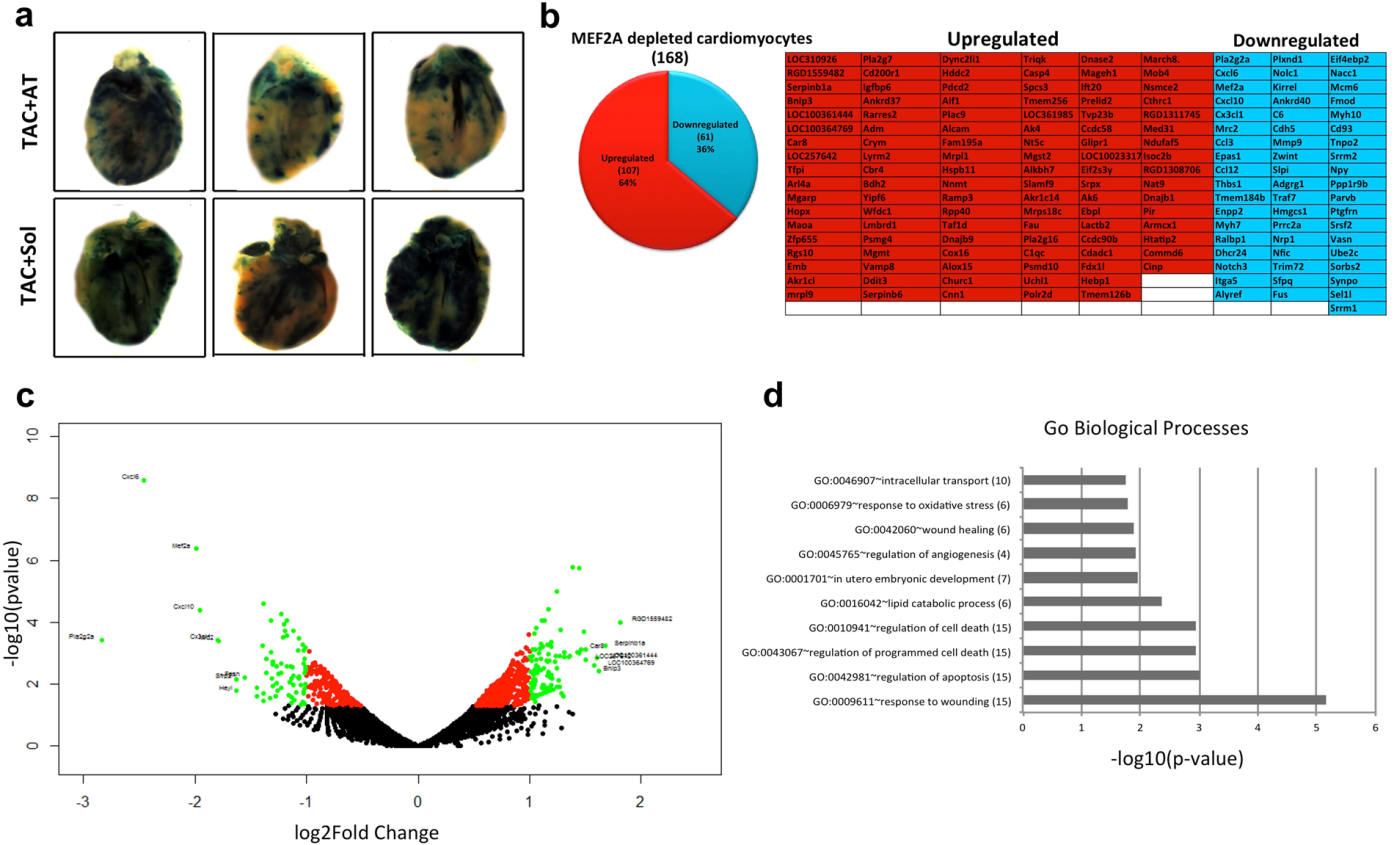
Involvement of MEF2 in cardiac hypertrophy and death. (**a**) Whole heart LacZ staining of MEF2-LacZ mice after TAC + Sol or TAC + AT treatment (n = 3). (**b**) The number of up or downregulated genes in response to MEF2A knockdown in primary cardiomyocytes. A table of genes identified by RNA-seq analysis after MEF2A knockdown (upregulated genes are in red; downregulated genes are in blue). (**c**) A volcano plot depicting up and downregulated genes identified in RNA-seq in response to MEF2A knockdown in primary cardiomyocytes. Black if p-value > 0.05, red if p-value < 0.05, green if log2FoldChange >1 or <−1 and p-value < 0.05. Labels are present only on logFC > ± 1.5. (**e**) GO Biological Processes associated with MEF2A knockdown in primary cardiomyocytes. The number of genes is in parentheses beside each term.

To determine how TAC and Atenolol influence MEF2 activity, MEF2 sensor mouse hearts were stained for LacZ expression. These data indicate that MEF2 activity was strongly induced in TAC + Sol mice and, in parallel with the positive reversal of other TAC induced changes, Atenolol treatment substantially reduced MEF2 activity (Fig. 5a). We then assessed potential regulation of these differentially expressed genes (429) from the TAC + AT vs TAC + Sol analysis using oPossum 3.0 to find transcription factor consensus sequences within ±5 kb of the transcription factor start site (data not shown). MEF2A consensus sequences were identified at 159 (37%) of differentially expressed genes. Next, we assessed predicted MEF2 consensus sequences from 32 overlapping genes upregulated in Fig. 2d. RNA expression (FPKM) of these genes was compared to results obtained in heart muscle (from Females, age 40–50) that were part of the Human Protein Atlas program (Supplementary Fig. S6). MEF2A, as a positive control, with FPKM >20 is highly expressed (+++) in heart muscle in this dataset. In comparison, RNA levels (FPKM), *Rarres2*, *Junb* and *Slc41a3* (++) have high heart muscle expression and *Aldh1a2*, *Ifrd1*, *Klf2*, *Leo1* have lower expression (+).

Since MEF2 activity was potently regulated by TAC and AT we next took a more reductionist approach by using siRNA-mediated MEF2A gene silencing to determine whether genes affected in the *in vivo* experiments were also influenced by MEF2A suppression in primary isolated cardiomyocytes. We reasoned that this approach might provide insight into the number of direct cardiomyocyte DEGs that were altered by β-adrenergic blockade that were a consequence of altered MEF2 function. siRNAs targeting MEF2A were transfected into primary cardiomyocytes and prepared for RNA-sequencing in duplicate. In these data we documented 168 DEGs, of which 107 were upregulated and 68 were downregulated with siRNA MEF2A depletion (Fig. 5b and Dataset S3). Figure 5c demonstrates the log2FoldChange of the differentially expressed genes in a volcano plot. The overall features of this plot reveal an extensive re-patterning of cardiomyocyte gene expression when MEF2A is depleted. Of note, *Mef2a* downregulation was coincident with downregulation of cytokines *Cxcl6* (rat homologue to mouse *Cxcl5*) and *Cxcl10* while *Bnip3*, which is involved in apoptotic induction, was upregulated. We have previously identified a protective role of MEF2A in cardiomyocyte survival^52^. It will be interesting to determine if upregulation of *Bnip3* is involved in apoptotic induction when MEF2A is silenced, a condition that does occur with acute β-adrenergic stimulation^53^.

Next we assessed the biological significance of the function of these genes using Gene Ontology analysis. Figure 5d shows the ten most enriched Biological Processes associated with MEF2A depletion. Interestingly, a number of these terms are related to cell death and the inflammatory response.

### *Rarres*2 is a novel MEF2 target gene regulated by β-adrenergic signaling

**Figure 6:**
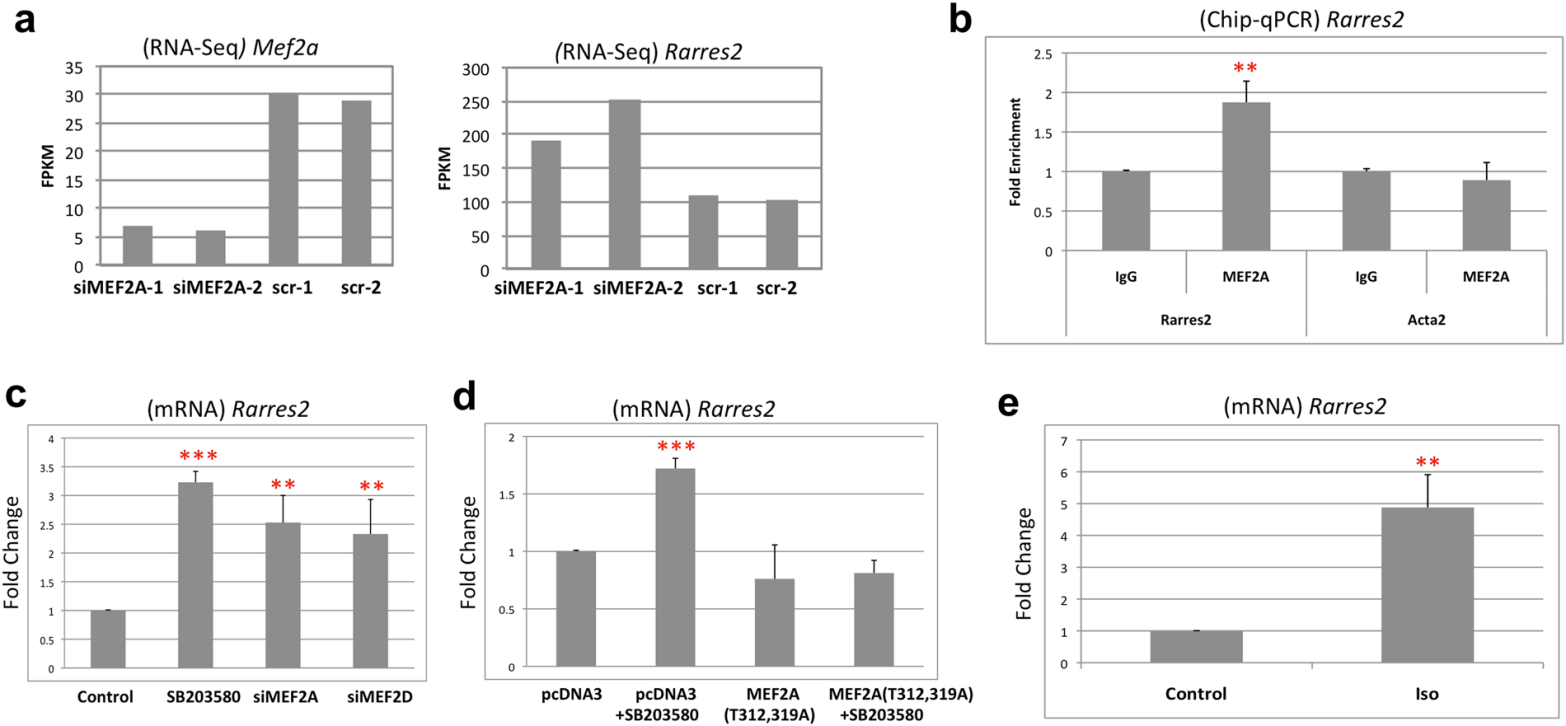
*Rarres2* is regulated by MEF2. (**a**) Expression of *Mef2a* and *Rarres2* in RNA-seq analysis of MEF2A depleted cardiomyocytes (fragments per kilobase of exon per million fragments mapped). (**b**) ChIP-qPCR analysis demonstrates that MEF2A is recruited to the *Rarres2* promoter in primary cardiomyocytes. Data are presented as fold enrichment (n = 3, **P < 0.01). Acta2 was used as a negative control. (**c**) *Rarres2* expression is negatively regulated by MEF2 in a p38 MAPK dependent manner. Primary cardiomyocytes were transfected with siRNA targeting MEF2A, MEF2D or a scrambled siRNA control and treated with SB 203580 (5 μM) for 24 hours. Control cells were treated with an inactive analogue, SB 202474. Data were normalized to Gapdh and are presented as fold change using the delta delta Ct method (n = 3, **P < 0.01, ***P < 0.001). (**d**) Phosphorylation defective mutated MEF2A prevents Rarres2 induction by p38 inhibition. Primary cardiomyocytes were transfected with empty vector (pcDNA3) or a mutated MEF2A expression construct (T312, 319A) and treated with SB 203580 or SB 202474 (5 μM) for 24 hours. Data were analyzed as in 6c (n = 3, **P < 0.01). (**e**) Isoproterenol (Iso; 10 μM) treatment for 24 hours, upregulates *Rarres2* expression. Data were normalized to Gapdh and are presented as fold change as in 6c (n = 3, **P < 0.01).

Supplementary Figuresranscriptome profiles from Fig. 2d to those affected by MEF2A knockdown *in vitro* we were particularly interested in the identification of the *Rarres2* mRNA. This gene encodes a protein for the adipokine, Chemerin. Previous studies have shown that treatment of cardiomyocytes with Chemerin affects Caspase 9 activity and induction of apoptosis^54^. The expression (FPKM) of *Mef2a* and *Rarres2* from the RNA-seq data is shown in Fig. 6a indicating efficacy of the gene silencing and an upregulation of *Rarres2*. This effect on Rarres2 in RNA levels is confirmed by RT-qPCR data in Fig. 6c and Supplementary Fig. S8. From our *in silico* analysis using oPossum, we found a putative MEF2 consensus sequence approximately 1 kb upstream from the transcription start site. Using primers flanking this region we performed ChIP-qPCR and validated MEF2A recruitment in primary cardiomyocytes to this locus (Fig. 6b, p < 0.01). We also determined that loss of MEF2A or MEF2D had a similar effect on *Rarres2* induction (Fig. 6c, p < 0.01). Figure 6c depicts cardiomyocyte treatment with the p38MAPK inhibitor (SB 203580) for 24 hours which resulted in an upregulation of *Rarres2* expression (p < 0.001). This effect is reduced when cardiomyocytes were transfected with a mutated MEF2A (MEF2A-T312, 319A) which is refractory to p38MAPK phosphorylation (Fig. 6d, p < 0.001). These data are consistent with suppression of *Rarres2* expression by p38MAPK-MEF2 signaling. In support of this, we observed that *Rarres2* is upregulated in cardiomyocytes by β-adrenergic signalling activation which results in MEF2 inhibition (Isoproterenol  treatment-Fig. 6e), similar to what was observed with TAC treatment *in vivo* (Fig. 2, Fig. 4a). Since we observed that exogenous *Rarres 2* expression resulted in cardiomyocyte hypertrophy in HL-1 cells (Fig. 4b) we next treated these cells with commercially available purified Chemerin peptide (100 nM) for 24 hours. This experiment resulted in a trend towards an increase in cell surface area, as an index of cellular hypertrophy (Supplementary Fig. S9). Further work needs to be carried out to determine the *in vivo* relevance of the *Rarres 2* gene in cardiac pathology since it is induced in experimental heart failure, is a target of p38 MAPK/MEF2 signaling and provokes a hypertrophic response when expressed in cultured cardiomyocytes.

## Discussion

Understanding the global gene networks associated with heart failure will provide critical insight into the underlying aetiology of heart disease while also providing an unprecedented array of molecular targets for therapeutic intervention. In the work presented here we have used next generation RNA sequencing based approaches coupled with bioinformatics to document transcriptome changes associated with experimental heart failure. Moreover, we have documented gene expression changes mediated by β–adrenergic antagonism that result from a partial reversal of a heart failure associated gene signature that is coupled with improved cardiac function.

Underlying the transcriptome changes that we have observed are dynamic changes in the activity of a fundamental cardiac transcriptional regulator, MEF2. The potent influence of β-adrenergic blockade in regulating MEF2 function in the heart has been highlighted recently^55^. However, the influence of β-blockers on MEF2 function in the heart is complex and requires attention to context since MEF2 responsiveness to β-blockade depends on the temporal nature of the treatment. Firstly, short term β-blockade leads to an acute upregulation of MEF2 activity^55^. Mechanistically this is well understood since β-adrenergic signalling promotes PKA activity and HDAC nuclear retention^52^. Both of these have strong repressive effects on MEF2 activity since PKA phosphorylates two residues in MEF2D (S121/190) that repress MEF2 function and HDAC retention in the nucleus enhances the formation of a MEF2/HDAC repressor complex^28^. We have recently reported that acute β-blockade essentially reverses this effect and upregulates MEF2 activity promoting a pro-survival function of MEF2 in cardiac myocytes that has potentially important clinical implications, particularly after myocardial infarction^55^. In support of this idea, we and others recently documented that experimental suppression of MEF2 expression in rodent cardiomyocytes using siRNA technology promotes apoptosis^55, 56^. Conversely, as we have shown here and somewhat unexpectedly, MEF2 activity under conditions of chronic (>4 weeks) β-adrenergic blockade leads to a downregulation of MEF2 activity, particularly under conditions where the heart is stressed as exhibited under conditions of TAC in mice. In our experiments long term (>6 weeks) TAC in mice leads to cardiac hypertrophy, progression towards heart failure, and pronounced activation of MEF2 activity. Treatment of mice at 6 weeks after TAC by four additional weeks of β-adrenergic blockade reduces the adverse impact on heart function and concomitantly suppresses chronic MEF2 activity while also invoking major transcriptome changes. In view of reports that MEF2 is a fundamental regulator of cardiac hypertrophy^10, 15^ and our results documenting the transcriptomic effects of MEF2 suppression in cardiomyocytes, we suspect that alterations in MEF2 activity are a major determinant of the transcriptome changes observed in response to TAC and also the positive outcome associated with β-adrenergic blockade.

Of particular interest in our studies are the gene expression changes that are induced by experimental heart failure and subsequently reversed by β-blockade since, in our view, some of these genes most likely mediate the protective effects of β-blockers in the heart. Cardiac hypertrophy is initially an adaptive compensatory mechanism to maintain cardiac output, however, prolonged hypertrophic conditions such as those seen during hypertension ultimately contribute to the progression towards heart failure^57^. Although β–blocker treatment in humans post-MI is undoubtedly effective in preventing or slowing the progression to heart failure, the molecular mechanisms involved are unclear. Here we provide the first steps in characterizing the molecular genetic basis of beneficial β–adrenergic antagonists in the heart. We report the identification of 32 genes that are differentially regulated by TAC whose expression pattern is reversed when TAC mice are treated with β-blockers. We further explored the role of *Junb*, *Alas2*, *Klf2*, and *Rarres2* as they were induced by TAC *in vivo* and their expression was repressed by Atenolol (Fig. 4a). Two of these (Junb and Klf2) are transcription factors whereas Alas2 regulates heme synthesis and Rarres2 (which encodes Chemerin) is an adipokine. Interestingly, these four different genes may potentiate different aspects of heart failure including oxidative stress, hypertrophy, cardiac remodelling, vascular remodelling and apoptosis. *Alas2* was shown to be upregulated in failing human hearts, resulting in oxidative stress and cell death^58^. However, in our *in vitro* experiments loss of *Alas2* in primary cardiomyocytes did not reduce apoptosis (Supplementary Fig. S7), indicating that Alas2 may promote cardiomyocyte death through alternate pathways. The transcriptional regulator, Junb, is directly responsible for the upregulation of MMp’s which mediate cardiac remodelling in ischemia-reperfusion studies^59^ and therefore reversal of its upregulation in pathological conditions may be critical. *Klf2* has been shown to be activated in endothelial cells by shear stress^60^. In our TAC model, constriction of the aorta results in increased hemodynamic stress, and likewise results in *Klf2* induction which is reversed by Atenolol. The role of KLF2 clearly requires further characterization in a heart context. Lastly, *Rarres2* was also upregulated in MEF2A knockdown cardiomyocytes *in vitro*, and has been previously associated with cardiac apoptosis and coronary artery disease^54^. It would be of interest to study β-adrenergic and MEF2 regulation of *Rarres2* in more detail in human heart disease. Taken together, these data indicate a potential involvement of these genes in the failing mouse heart that may have implications in human heart failure. Furthermore, the identification of genes associated with heart failure that are downregulated by Atenolol (such as *Junb, Klf2, Alas2*, and *Rarres2)* demonstrates the underlying molecular changes responsible for improved heart function, which may lead to new downstream targets for treating heart failure.

While the strength of the current study is the unbiased identification of genes in the heart that are dysregulated during heart failure and, importantly, those that respond to drug treatment, it is nevertheless important to emphasize that these data provide a platform for further study. Clearly in the present study it is not possible to ascertain which of the genes in these large cohorts of DEG’s are protective in the heart versus those whose expression pattern is merely correlated with the changes in function. By nature, high throughput analysis is largely descriptive, thus requiring extensive downstream analysis to determine cause and effect relationships between the genes identified and the biological process(es) under study. While we have begun to do this in the current study in a limited way, a detailed analysis of so many genes and their function in heart failure will take many years. Moreover, the functional adaptations, for example with β-blocker treatment, likely result from changes in expression of multiple genes. To systematically study the cause and effect nature of whole cohorts of genes in a compound manner is currently very challenging, especially *in vivo*, and may require further advances in technology and possibly computational predictive approaches. An additional limitation of the current study is that it represents an endpoint analysis of changes in gene expression as result of the various treatments. Although this alone has been an extensive task, it would be instructive to have a time course analysis of changes in gene expression across the experimental conditions, which may identify important transitional patterns of gene expression at different stages of the pathology and treatment. Lastly, we would point out that additional work to address whether the changes at the RNA level (by RNA seq and RT-qPCR) are reflected in the proteome is also necessary. This might be achieved using state of the art quantitative proteomics approaches in parallel with transcriptome studies.

Overall, we contend that the cohort of DEGs reported here represent vast potential for the diagnosis and treatment of heart disease. Interestingly, we have noted the identification of genes and GO processes associated with the immune system. These observations support recent developments reflecting the involvement of the immune system and inflammatory responses in the progression of heart disease^61–63^. It will be of interest to further analyze these inflammatory related protein coding genes to determine if they do indeed contribute to heart disease. Herein, we report broad categories of biological processes and also individual genes that are differentially regulated under conditions of experimental heart failure that can be reversed by pharmacological treatment with β-adrenergic antagonists. The next steps will be to determine whether these changes are mirrored in human heart disease and to develop a flexible array of therapeutics that can detect and potentially target these processes with the ultimate aim of reducing pathological changes in the myocardium, enhancing cardiac function, and improving patient morbidity and mortality associated with heart disease.

## Methods

### Induction of pressure overload by transaortic constriction (TAC) in mice

Six to eight week old MEF2-LacZ transgenic male sensor mice, reported previously^34^, were used in this study. Under general anesthesia (i.p. xylazine: 0.03 mg/g; ketamine: 0.15 mg/g), hair from the chest was removed and the surgical area disinfected with betadine. A skin incision was made along the midline from the neck to the rib cage and the chest cavity was opened. The rib cage and thymus were retracted to expose the transverse aorta. A 27g needle was used to calibrate a microclip applicator. A titanium microligation clip was applied between the origins of the innominate and left common carotid arteries, constricting the transverse aorta to the gauge of the needle. The rib cage, muscles, and skin were closed with a 6–0 USP non-absorbable silk suture. The animals were then administered s.c. 0.03 μg/mg Buprenorphine and allowed to recover on a heating pad until fully awake. Sham surgeries were performed as above except the microligation was not applied to the transverse aorta. All mice were monitored after the procedure for normal behaviour in accordance with Canadian Council on Animal Care regulations.

### Atenolol administration *in vivo* and β galactosidase staining

β-blockers were administered through drinking water (Atenolol; 50mg/kg/day) or Solvent (5 ml water) for 4 weeks as indicated. Mice were sacrificed by cervical dislocation and the apex of each heart was fixed with 2% paraformaldehyde in PBS for 30 minutes. The samples were washed three times with PBS, and incubated at 37 °C with X-Gal (5-bromo-4-chloro-3-indolyl-β-d-galactopyranoside) staining solution (5 mM ferrocyanide, 5 mM ferricyanide, 2 mM MgCl_2_, and 1 mg/ml X-Gal) to visualize β-Gal positive cells. Then all the samples were examined using bright field microscopy.

### Analysis of cardiac function using high-frequency ultrasound imaging technology

All mice were subjected to transthoracic echocardiographic analysis to measure heart function 6 and 10 weeks following aortic banding or sham surgery. Cardiac function and heart morphology were evaluated using echocardiography (Vevo 2100, VisualSonics). The animals were sedated using 3% isoflurane and maintained with 1~2% isoflurane. The parasternal long axis view (B-mode, M-mode) was obtained and measurements of cardiac structure and function were determined as described previously^64^. The individuals performing echocardiographic analysis of heart function were blinded to surgical group and genotype.

### Cardiac tissue collection, H&E, Masson’s Trichrome, and wheat germ agglutinin staining (WGA) staining

Following echocardiography, mice were weighed and euthanized using cervical dislocation. Hearts were excised and quickly perfused with 30mM KCl to induce diastolic arrest. Hearts were then weighed and divided for further analysis. Mid-ventricular cross-sections of freshly dissected heart tissue were fixed in 10% formalin solution for 1 hour then stored in 70% ethanol at 4oC until further processing. Fixed heart tissues were dehydrated to xylene and embedded in pure paraffin wax blocks. Paraffin-embedded sections were deparaffinized and rehydrated first with descending concentrations of ethanol and then brought into double distilled water. The sections were then subjected to H&E or Masson’s Trichrome staining per manufacturer’s instructions, or incubated with wheat germ agglutinin (WGA; Alexa Fluor 488 conjugated) for 2 hours in the dark, briefly washed with PBS and then mounted on microscopy slides with ProLong Gold. Fluorescent images were captured with an Olympus confocal microscope and analyzed with NIH ImageJ software (v.147). Cardiomyocyte cross-sectional area was determined by manually tracing the cell membrane of a minimum of 100 cardiomyocytes from representative triplicate experiments using NIH ImageJ software (v.147). To quantitate fibrosis, five animals per experimental group were analyzed. Three representative images per animal were imported into ImageJ, converted into RGB stacks using the k-means clustering extension, then limited to pixels within the selected threshold intensity range to select areas of fibrosis (blue colour) as a function of % area on the red channel.

### Primary cardiomyocyte isolation

Primary neonatal rat cardiomyocytes were prepared from 1- to 3-day old rats (Sprague Dawley) using the Neonatal Cardiomyocyte Isolation System (Worthington Biochemical Corp). Neonatal rat pups were sacrificed by decapitation. In brief, whole hearts were dissociated and digested with trypsin (Promega) and collagenase (Worthington Biochemical Corp). Cardiomyocytes were re-suspended in culture medium with (DMEM/F12 (Gibco) with 10% fetal bovine serum (FBS), 1% Penicillin/Streptomycin and 50 mg/L gentamycin sulphate). The isolated cells were plated on 10 cm dishes for 60 minutes in 37 °C humidified incubator with a 5% CO_2_ in air to remove non-myocardial cells. The cardiomyocytes were then seeded into gelatin-coated 6-well plates. The day after, medium was removed and replaced by fresh medium.

### Cardiomyocyte transfection

Neonatal cardiomyocytes were transiently transfected with siRNA using Lipofectamine RNAiMAX (Invitrogen). For each well, according to the manufacture’s instruction, Lipofectamine RNAiMAX reagent was diluted into 150 μl in Opti-MEM medium, and in a separate tube, siRNA (100nM) was also diluted in 150 μl Opti-MEM medium, mixed and incubated for 5 minutes at room temperature. The 250 μl of siRNA/Lipofectamine mixture was added to cells and incubated at 37 °C overnight. Following the incubation, media was replaced and harvested 48 hours later for experimental procedures. The siRNAs purchased from Sigma Aldrich were, siMEF2A#1 (SASI_Mm01_00120787) or scrambled control (SIC001). The siRNA purchased from Thermo Fisher Scientific were, siRarres2 (ID: 252512, ID: 252511), siJunb (ID: 59715, ID: 51454), siAlas2 (ID: 468000, ID: 46614), siKLF2 (ID: 218080, ID: 218079) and siIigp1 (ID: 76220, ID: 76128). Isoproterenol hydrochloride (Sigma Aldrich, 1351005) (10 µM) was used to induce apoptosis in primary cardiomyocytes. Caspase 3 (Cell signaling, 9662) and cleaved Caspase 3 antibodies (Cell signaling, 9661) were used for western blot analysis and to measure apoptosis a ratio of cleaved Caspase 3 to total Caspase 3 was used. The westerns were run on an odyssey quantitative blotting system so the quantitation is linear.

### HL1 Cell Culture and Transfection

The HL-1 cardiac cell line was cultured in Claycomb Medium (SigmaAldrich) supplemented with 100 μM norepinephrine (Sigma Aldrich), 10% FBS and 4 mM L-glutamine (Invitrogen). Cells were maintained in a humidified 37 °C incubator with 5% CO_2_. The HL-1 cell line was originally established from an AT-1 subcutaneous tumor excised from an adult female Jackson Laboratory inbred C57BLy6J mouse. Transient transfections were performed using lipofectamine 2000. A 1:2.5 mixture ratio of DNA to lipofectamine in 250 μl Opti-Medium (Gibco) was prepared and incubated overnight. The following day media was removed and replaced with fresh media. The HL-1 transfected cells were incubated with wheat germ agglutinin (WGA; Alexa Fluor 637 conjugated) for 2 hours in the dark, briefly washed with PBS and then mounted on microscopy slides. Recombinant human Chemerin protein (Abcam) (100 nM) was added to HL-1 cultures for 24 hrs and WGA staining was performed as above. Fluorescent images were captured with an Olympus confocal microscope and analyzed with NIH ImageJ software to determine cell cross sectional areas.

### RNA preparation for sequencing

Total RNA was isolated from the mouse left ventricle or primary rat cardiomyocytes using the Qiagen Universal RNA kit. Five μg of RNA was sent to the McGill University and Genome Quebec Innovation Centre (MUGQIC) for cDNA library preparation (Illumina Truseq mRNA kit). Paired reads were generated using Illumina HiSeq 2000 sequencer (100 bp paired-end reads). RNA-seq data has been deposited in the NCBI’s Gene Expression Omnibus (GEO) accession number: GSE75213

### Differential expression analysis of transcripts from TAC+AT experiments

For mRNA, mouse reads were aligned to mm10 as previously reported^65^. Differentially expressed mRNA transcripts were filtered and identified with edgeR, using the following options: i) Transcripts are represented by 5 or more reads per million in at least 6 among the 12 experiments. Transcripts are identified as differentially expressed in 5 comparisons ii) Transcripts which are up-or downregulated by 50%, for a False-Discovery-Rate (FDR) of 20% or less (in the output of edgeR’s glmLRT function) are selected. Clustering heatmaps were generated with Matlab’s Bioinformatics toolbox. Primary miRNA transcripts were identified using edgeR (adjusted p-value < 0.2). To find lncRNA, short paired-end reads were mapped with RSEM/Bowtie to the reference index (mm9) which was built for the 2073 mouse lncRNAs^66^. All uniquely mappable short reads are included by using the bowtie-m 1 option in the rsem-calculate-expression command. For identifying differentially expressed lncRNAs we used options that differed slightly from the analysis of protein-encoding transcripts: i) Transcripts are represented by 5 or more reads per million in at least 5 among the 12 experiments. ii) Reads were multiplied by coefficients assuming equal library sizes for each of the 12 experiments. iii) A two-factor generalized linear model design matrix was specified to estimate dispersion parameters. The Huber function with k = 1.03 was used for robust statistics in the estimation of the tagwise dispersion parameters by the edgeR software^67^. LncRNAs are identified as differentially expressed in 5 different comparisons and by using a False-Discovery-Rate (FDR) cut-off of 20% in the output of edgeR’s glmLRT function.

### Differential expression analysis of transcripts in primary rat cardiomyocytes

NGS short reads were mapped to the rat genome with the RSEM/Bowtie software (Dewey Lab) and an index generated for 17349 mature transcripts in Rnor_6 coordinates. Raw counts were analysed with edgeR using similar identical parameters as in above: requiring that transcripts are represented by 5 or more reads in at least 3 out of 4 experiments (2 siMEF2A replicates and 2 controls). In the MEF2A-depleted cardiac cells, 168 genes, excluding MEF2A, were significantly up- or downregulated, using an FDR of 25%.

### Bioinformatics

DAVID annotation clustering was done using default settings. Annotation terms were chosen from the list of representative terms generated within each cluster. If present, the first SP_PIR_KEYWORDS annotation was used to represent the annotation cluster. If SP_PIR_KEYWORDS was not present, the first Biological Processes (BP) term to occur in the cluster was used. The heatmaps and volcano plot were generated using R software. (https://www.r-project.org/). MEF2 consensus sequences were identified using oPOSSUM3.0 (http://opossum.cisreg.ca/oPOSSUM3/).

### Reverse Transcription Quantitative PCR

Total RNA was extracted using the RNeasy Plus Mini kit (Qiagen) according to the manufacturer’s protocol. RNA was converted to cDNA using Superscript III (Invitrogen) according to the manufacturer’s instructions. Note that cDNA was combined with iTaq universal SYBR Green super mix (Bio-Rad) and 500 nM primers in a final volume of 20 μl. Each sample was prepared in triplicate and analyzed using Rotor-GeneQ (Qiagen) according to the manufacturer’s protocol. All values were normalized to GAPDH (internal control) mRNA levels.

### Statistics

Data are reported as mean ± SEM. Independent two sample t-tests of all quantitative data were conducted, whereas a two-way analysis of variance followed by a Sidak’s multiple comparisons test was performed on experiments with more than one experimental condition.

P-value of <0.05 was considered statistically significant. All data were done on technical triplicates and biological triplicates. The n = 3 in the caption indicates the number of biological replicates.

### Study Approval

All mouse experiments were approved by the York University Animal Care Committee in accordance with Canadian Council of Animal Care regulations.

## Electronic supplementary material

Supplementary Figures

Data set 1

Dara set 2

Data set 3
