# Supplementary material for: Heart Failure and MEF2 Transcriptome Dynamics in Response to β-Blockers
Source: Sci Rep. 2017 Jun 30;7:4476. doi: 10.1038/s41598-017-04762-x (PMC5493616; doi:10.1038/s41598-017-04762-x)
Supplement: Supplementary file 1 — Supplementary Figures [file 41598_2017_4762_MOESM1_ESM.pdf]

Title: Heart Failure and MEF2 Transcriptome Dynamics in Response to β-Blockers.

Authors: *Tobin, S.W.[1,2,3], *Hashemi, S.[1,2,3], Dadson, K.1, Turdi, S. [1], Ebrahimian, K. [1,2,3], Zhao, J.[1,2,3], Sweeney, G.1, Grigull, J.[1,4], and # McDermott, J.C [1,2,3,5]

*contributed equally to the work, # corresponding author

[1] Department of Biology, York University, [2] Muscle Health Research Centre (MHRC), [3] Centre for Research in Biomolecular Interactions (CRBI), [4] Department of Mathematics and Statistics, York University, [5] Centre for Research in Mass Spectrometry (CRMS)

# To whom correspondence should be addressed:
John C. McDermott, PhD.
Department of Biology, York University
4700 Keele St.
Toronto, Canada M3J 1P3;
Phone: 416-736-2100 Ext. 30389;
Fax: 416-736-5698;
Email: jmcderm@yorku.ca

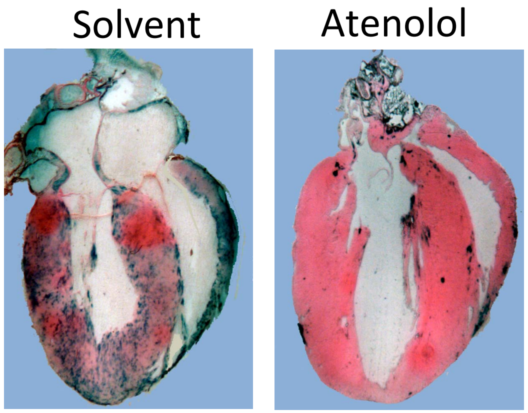

Supplementary Figure S1. MEF2-LacZ activity in heart with chronic 10 week β-blocker (Atenolol) treatment (50mg/kg/day).

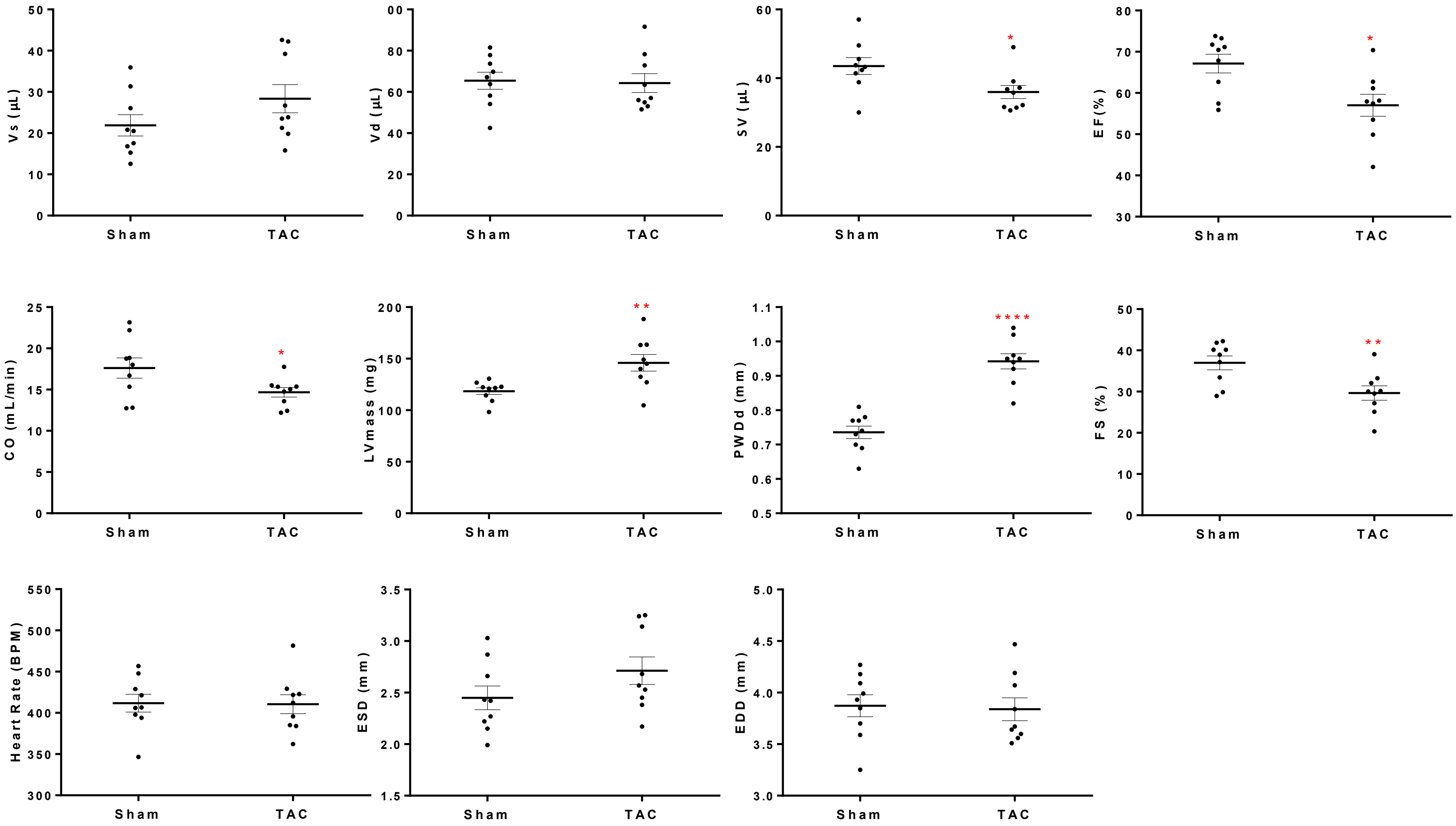

Supplementary Figure S2. Baseline echocardiography analysis before administration of Atenolol or solvent. Mean ± S.E.M (n=18), t-test was used, **P*<0.05 ***P*<0.01 *****P*<0.0001 *vs.* Sham+Sol.

a

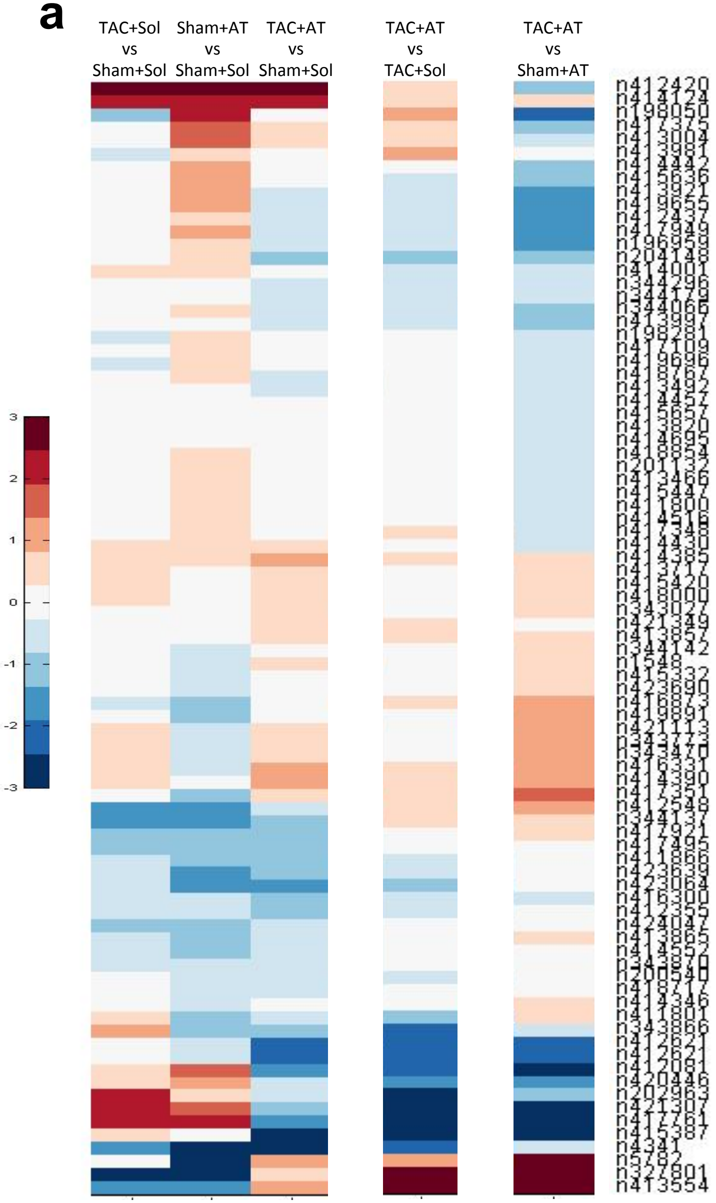

b

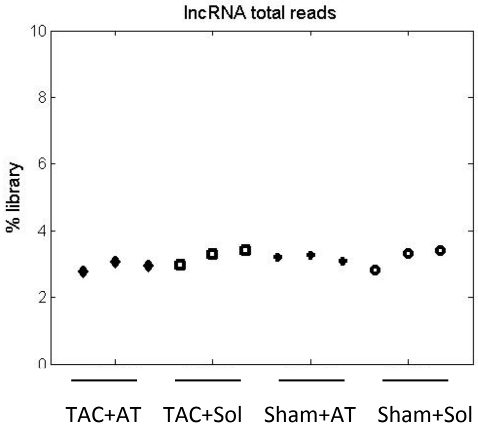

c

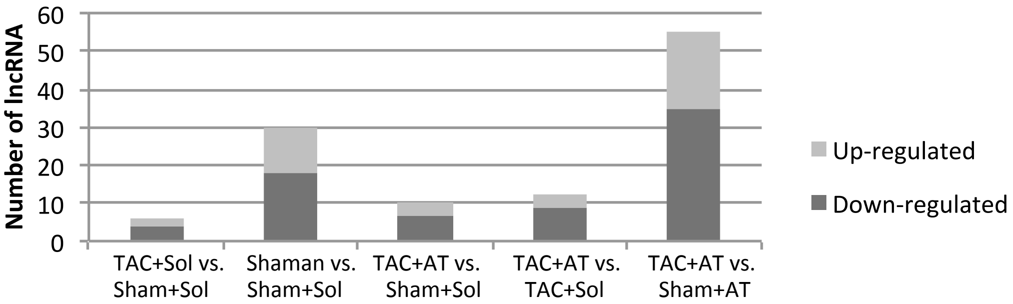

d

Sham+Sol	TAC+Sol	Sham+AT	TAC+AT	ncid	Name	Accession number
*	*	*	*	n413706	-	NULL
*	*	*	*	n415312	Mhrt	NR_033497
*	*	*	*	n413977	-	-
*	*		*	n413824	BC002163	NR_002445_2
	*	*	*	n413921	Gm6548	NR_003363
		*		n413820	-	-
*				n424047	MSUR1	AY248756

e

NONCODE ID (2016)	Strand	ncid v3	Expression Pattern	Co-ordinates (mm9)	Length	Ensembl
NONMMUT225116	-	n202963	up in TAC; down in TAC+AT	chr12: 14155665-14156393	1891	-
NONMMUT284543	+	n413554	down in TAC; up in TAC+AT	chr4: 145686274-145687162	1318	ENSMUST00000181610

Supplementary Figure S3. Heart failure associated changes in lncRNA expression. A mouse lncRNA database containing 2073 lncRNA genomic positions was obtained from Matkovich et al. (66). Short paired-end reads from 12 *in vivo* samples were mapped with RSEM/ Bowtie to the reference index which was built for the 2073 mouse lncRNAs (mm9). All uniquely mappable short reads are included by using the bowtie-m 1 option in the rsem-calculate-expression command. a) Significant fold changes for 84 lncRNA (FDR<0.2; red upregulated; blue downregulated). lncRNA are sorted by p-value based on the left-most column. The three columns in the left part of the figure show fold changes with respect to reference condition Sham+Sol. The next two columns show the significance of fold changes in TAC+AT with respect to reference conditions TAC+Sol and Sham+AT, respectively. b) The percentages of reads which mapped to lncRNAs varied in a narrow range from 2.8% to 3.4% c) The number of differentially expressed lncRNAs per condition. d) The five most abundant lncRNAs in each treatment. e) DE lncRNAs targeted in both TAC+Sol vs Sham+Sol and TAC+AT vs TAC+Sol.

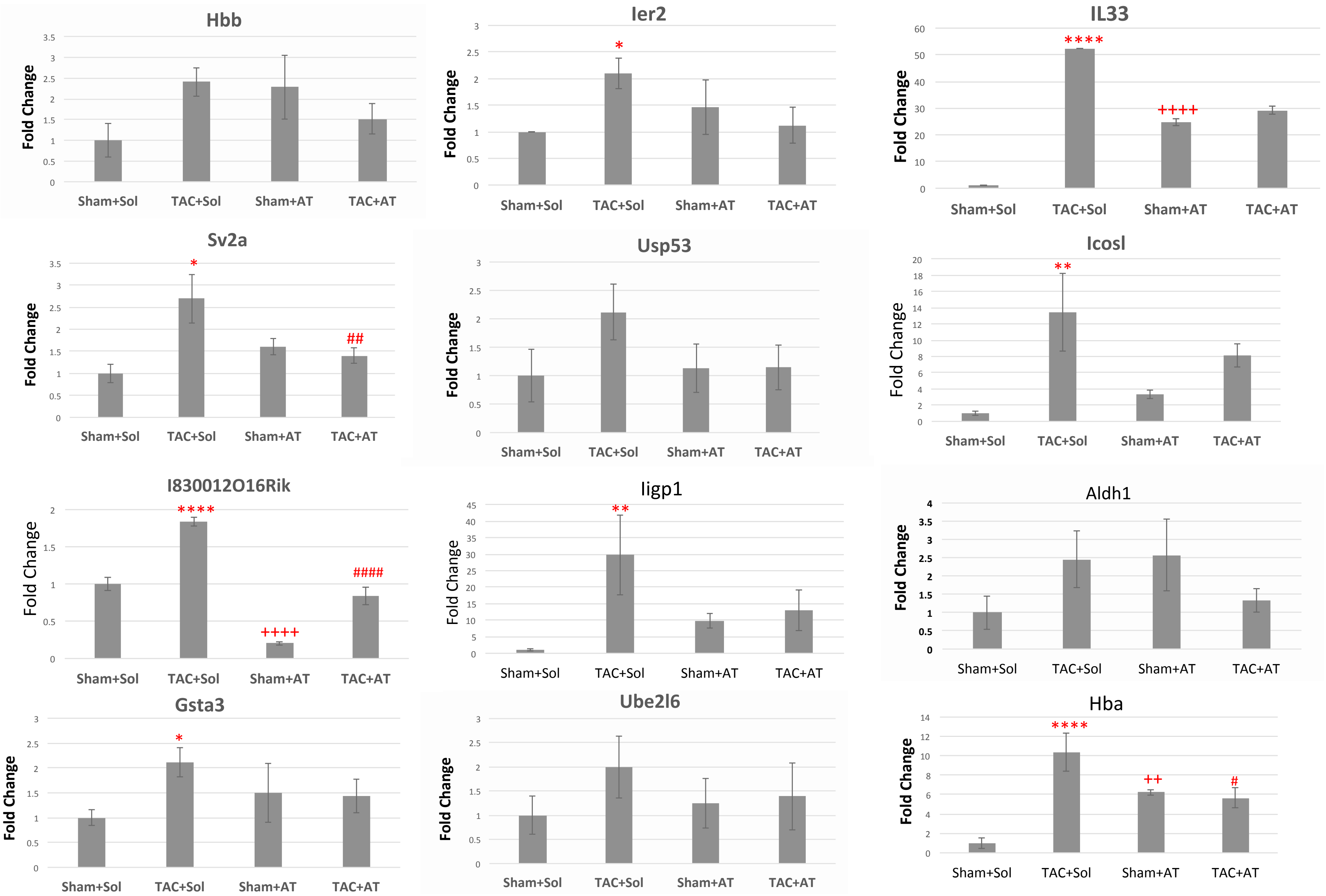

Supplementary Figure S4. Expression of genes upregulated in TAC and down-regulated with Atenolol treatment (Figure 2C), was confirmed using RT-qPCR. Data were normalized to Gapdh and are presented as fold change using the delta delta Ct method (n=3, *P<0.05 **P<0.01 **** P<0.0001 Sham+Sol vs TAC+Sol, ++P<0.01 ++++P<0.0001 Sham+Sol vs Sham+AT, #P<0.05 ##P<0.01 ####P<0.0001 TAC+Sol vs TAC+AT).

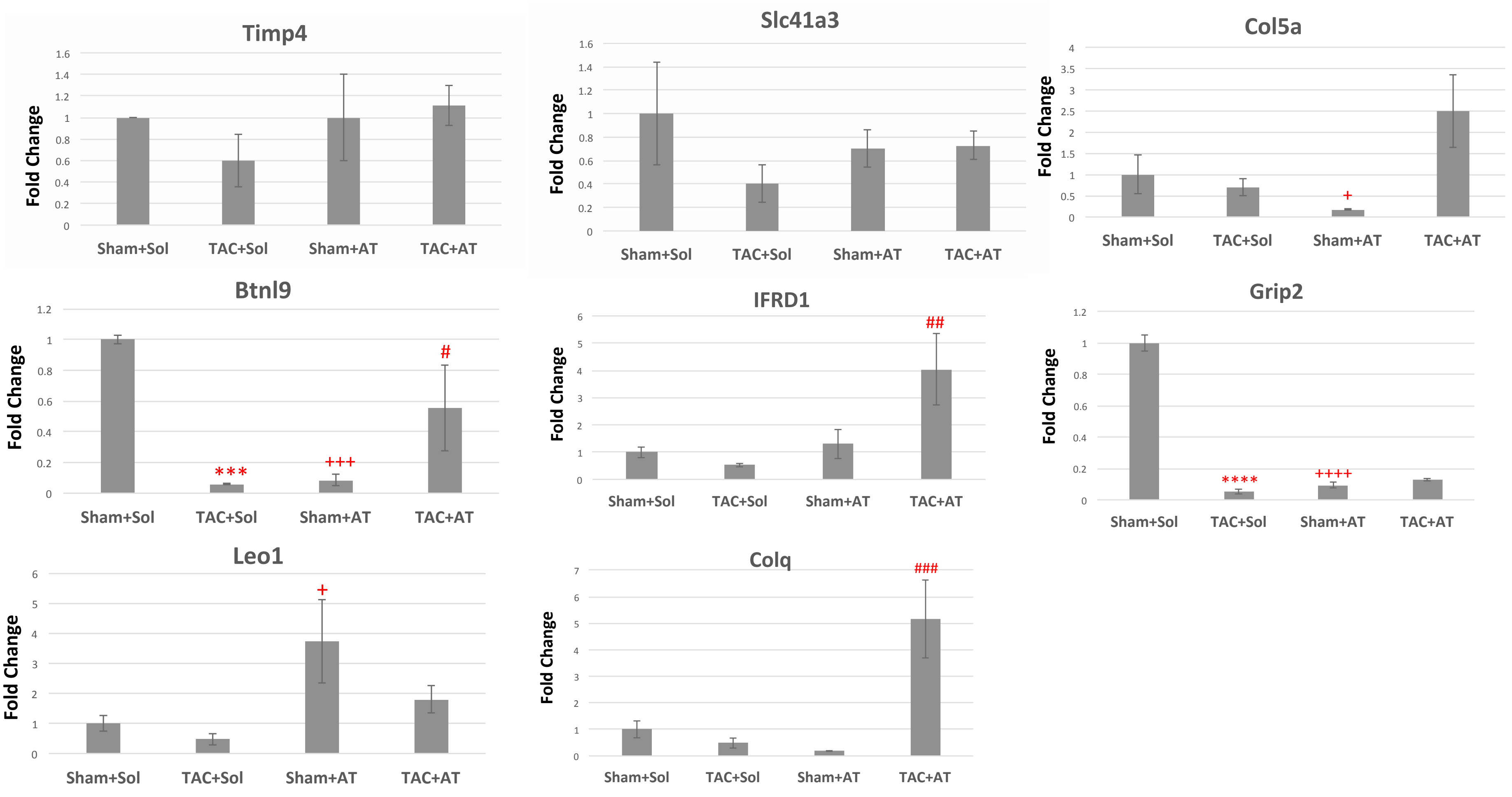

Supplementary Figure S5. Expression of genes down-regulated in TAC and upregulated with Atenolol treatment (Figure 2c), was confirmed using RT-qPCR. Data were normalized to Gapdh and are presented as fold change using the delta delta Ct method (n=3, ***P<0.001 ****P<0.0001 Sham+Sol vs TAC+Sol, +P<0.05 +++P<0.001 ++++P<0.0001 Sham+Sol vs Sham+AT, #P<0.05 ##P<0.01 ###P<0.001 TAC+Sol vs TAC+AT).

Gene ID	Gene Name	Ensembl ID(s)	Chr	Start	End	Strand	TFBS Rel. Start	TFBS Rel. End	Abs. Score	Rel. Score	TFBS Sequence	RNA level
ALDH1A2	aldehyde dehydrogenase family 1, subfamily A2	ENSMUSG00000013584	9	71063596	71144050	+	-4191	-4182	12.569	90.70%	CTATTTTTAT	+
							-4175	-4166	10.009	85.30%	CTCTTTATAA	
							-4	6	9.917	85.10%	CTATATACAG	
ifrd1	interferon-related developmental regulator 1	ENSMUSG00000001627	12	40928154	40975091	-	4593	4602	12.3	90.10%	TTATATATAG	+
junb	Jun-B oncogene	ENSMUSG00000052837	8	87500811	87502617	-	-3601	-3592	14.351	94.40%	CTATTTATAA	++
klf2	Kruppel-like factor 2 (lung)	ENSMUSG00000055148	8	74842932	74845555	+	-89	-80	10.213	85.80%	CTAAATTTAG	+
LEO1	Leo1, Paf1/RNA polymerase II complex component, homolog (S. cerevisiae)	ENSMUSG00000042487	9	75289331	75314239	+	-229	-220	13.587	92.80%	CTATTTTTAA	+
Iigp1b/iigp1	interferon inducible GTPase 1; interferon-inducible GTPase-like	ENSMUSG00000054072	18	60535683	60552281	+	-389	-380	12.436	90.40%	CTAATTTTAG	-
RARRES2	retinoic acid receptor responder (tazarotene induced) 2	ENSMUSG00000009281	6	48519697	48522669	-	-1031	-1022	11.561	88.60%	CTATCTTTAG	++
SLC41A3	solute carrier family 41, member 3	ENSMUSG00000030089	6	90554719	90596406	+	-2853	-2844	11.561	88.60%	CTATCTTTAG	++
SV2A	synaptic vesicle glycoprotein 2 a	ENSMUSG00000038486	3	95985074	95999444	+	1503	1512	14.079	93.80%	CTATATTTAG	-
MEF2A	Myocyte Enhancer Factor 2A											+++

RNA Levels (FPKM)
No expression (-)
Less than 10 (+)
Less than 20 (++)
More than 20 (+++)

Supplementary Figure S6. Predicted MEF2 consensus sequences from 32 overlapping genes in Figure 2D. RNA expression (FPKM) of these genes in heart muscle (Female, age 40-50) were analyzed in Human Protein Atlas program.

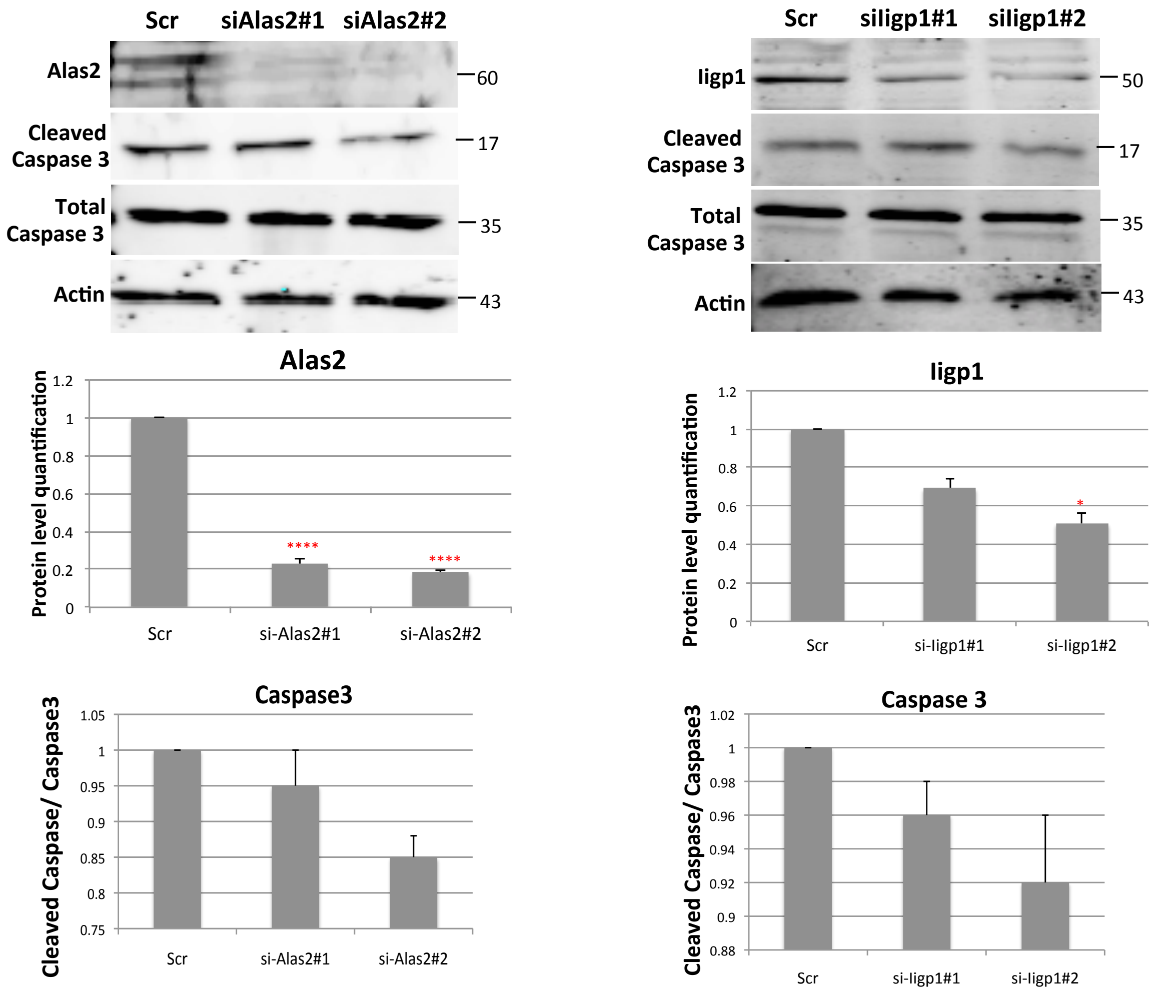

Supplementary Figure S7. Primary cardiomyocytes were transfected with two independent siRNAs, Alas2 siRNA and Iigp1 siRNA or with scramble siRNA control and treated with isoproterenol (10μM) for 48 hours. Equal amounts of total protein were used for western blot analysis and the levels of the indicated proteins were assessed by a standard immunoblotting technique using specific primary antibodies for each as indicated. (n=3, *P<0.05 ****P<0.0001).The western has been cropped. An odyssey quantitative blotting system is used for the western blot analysis, therefore the quantitation is linear.

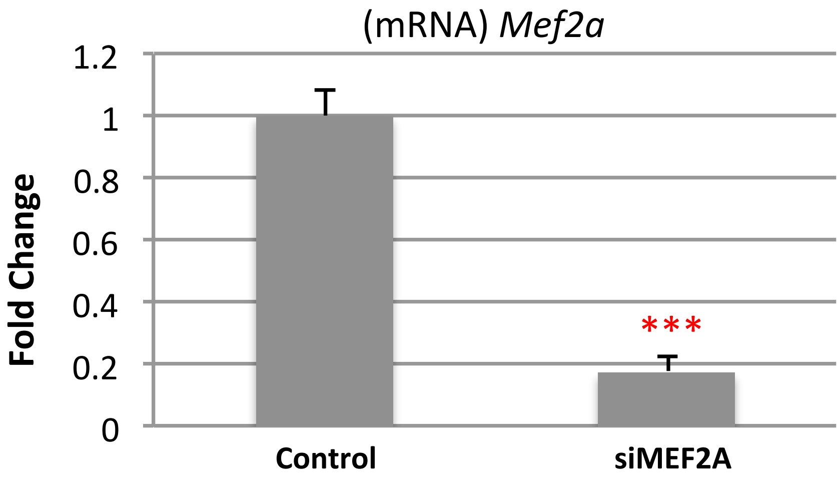

Supplementary Figure S8. Expression of *Mef2a* in MEF2A depleted cardiomyocytes is confirmed using RT-qPCR. Data were normalized to Gapdh and are presented as fold change using the delta delta Ct method (n=3, ***P<0.001).

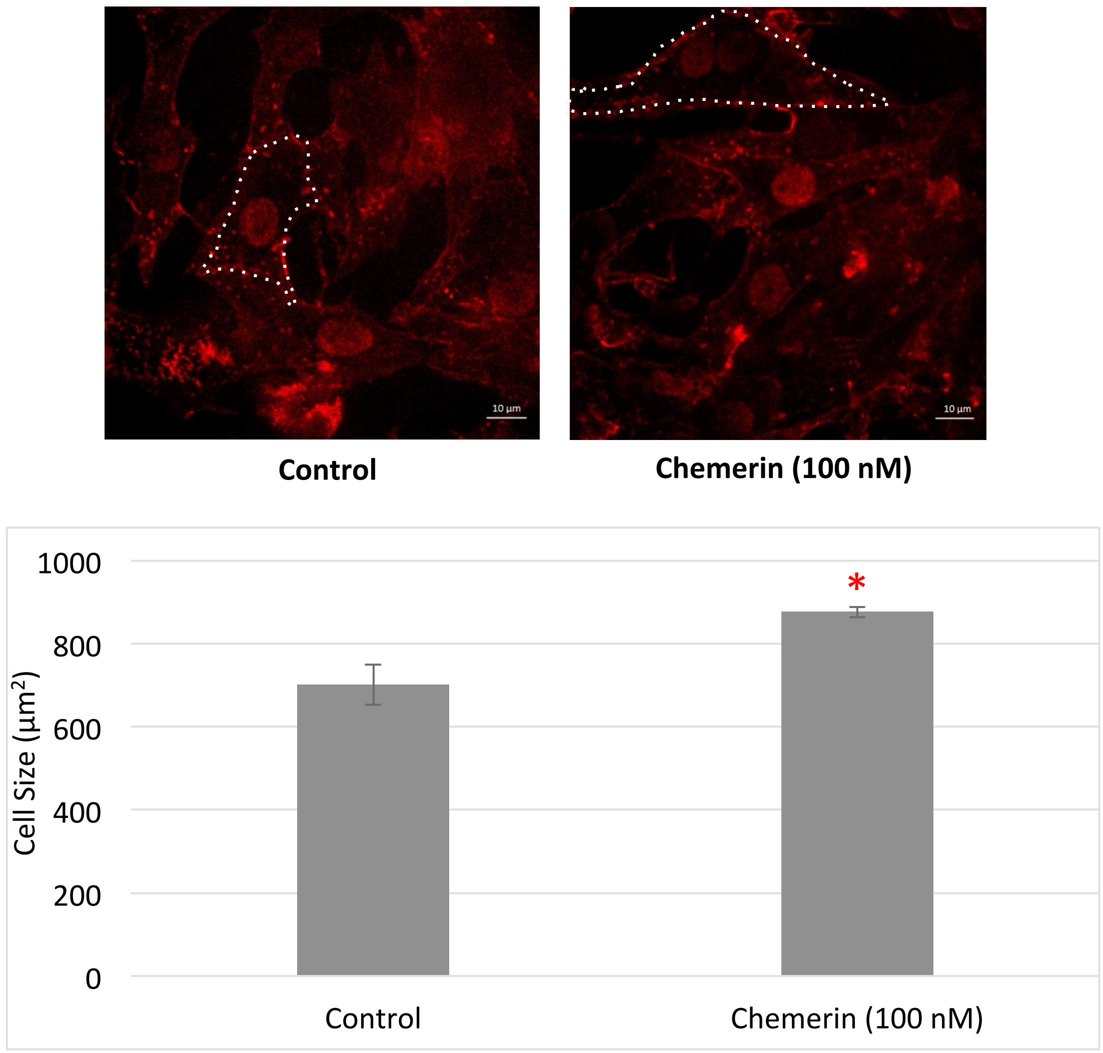

Supplementary Figure S9: Primary Cardiomyocytes were treated with Chemerin (100 nM) for 24 hrs and stained with Wheat Germ Agglutinin (WGA) in red. The bar graph indicates that cell size of cardiac cells, quantified based on 5 cell measurements per image. Data are presented, as mean ±SEM. *P<0.05. Scale bar is 10µm.
